# Integrative transcriptomic and metabolomic analyses unveil tanshinone biosynthesis in *Salvia miltiorrhiza* root under N starvation stress

**DOI:** 10.1371/journal.pone.0273495

**Published:** 2022-08-25

**Authors:** Li-Lan Lu, Yu-Xiu Zhang, Yan-Fang Yang

**Affiliations:** 1 Hainan Provincial Key Laboratory of Resources Conservation and Development of Southern Medicine, Hainan Branch of the Institute of Medicinal Plant Development, Chinese Academy of Medical Sciences & Peking Union Medical College, Haikou, China; 2 Hainan Key Laboratory of Tropical Oil Crops Biology/Coconut Research Institute, Chinese Academy of Tropical Agricultural Sciences, Wenchang, China; 3 State Key Laboratory of Tree Genetics and Breeding, Key Laboratory of Tree Breeding and Cultivation of State Forestry Administration, The Research Institute of Forestry, Chinese Academy of Forestry, Beijing, China; CSIR- Institute of Himalayan Bioresource Technology, INDIA

## Abstract

*Salvia miltiorrhiza* is a model plant for Chinese herbal medicine with significant pharmacologic effects due to its tanshinone components. Our previous study indicated that nitrogen starvation stress increased its tanshinone content. However, the molecular mechanism of this low nitrogen-induced tanshinone biosynthesis is still unclear. Thus, this study aimed to elucidate the molecular mechanism of tanshinone biosynthesis in *S*. *miltiorrhiza* under different N conditions [N-free (N0), low-N (Nl), and full-N (Nf, as control) conditions] by using transcriptome and metabolome analyses. Our results showed 3,437 and 2,274 differentially expressed unigenes between N0 and Nf as well as Nl and Nf root samples, respectively. N starvation (N0 and Nl) promoted the expression of the genes involved in the MVA and MEP pathway of tanshinone and terpenoid backbone biosynthesis. Gene ontology and KEGG analyses revealed that terpenoid backbone biosynthesis, hormone signal transduction, and phenylpropanoid biosynthesis were promoted under N starvation conditions, whereas starch and sucrose metabolisms, nitrogen and phosphorus metabolisms, as well as membrane development were inhibited. Furthermore, metabolome analysis showed that metabolite compounds and biosynthesis of secondary metabolites were upregulated. This study provided a novel insight into the molecular mechanisms of tanshinone production in *S*. *miltiorrhiza* in response to nitrogen stress.

## Introduction

**Fig 1:**
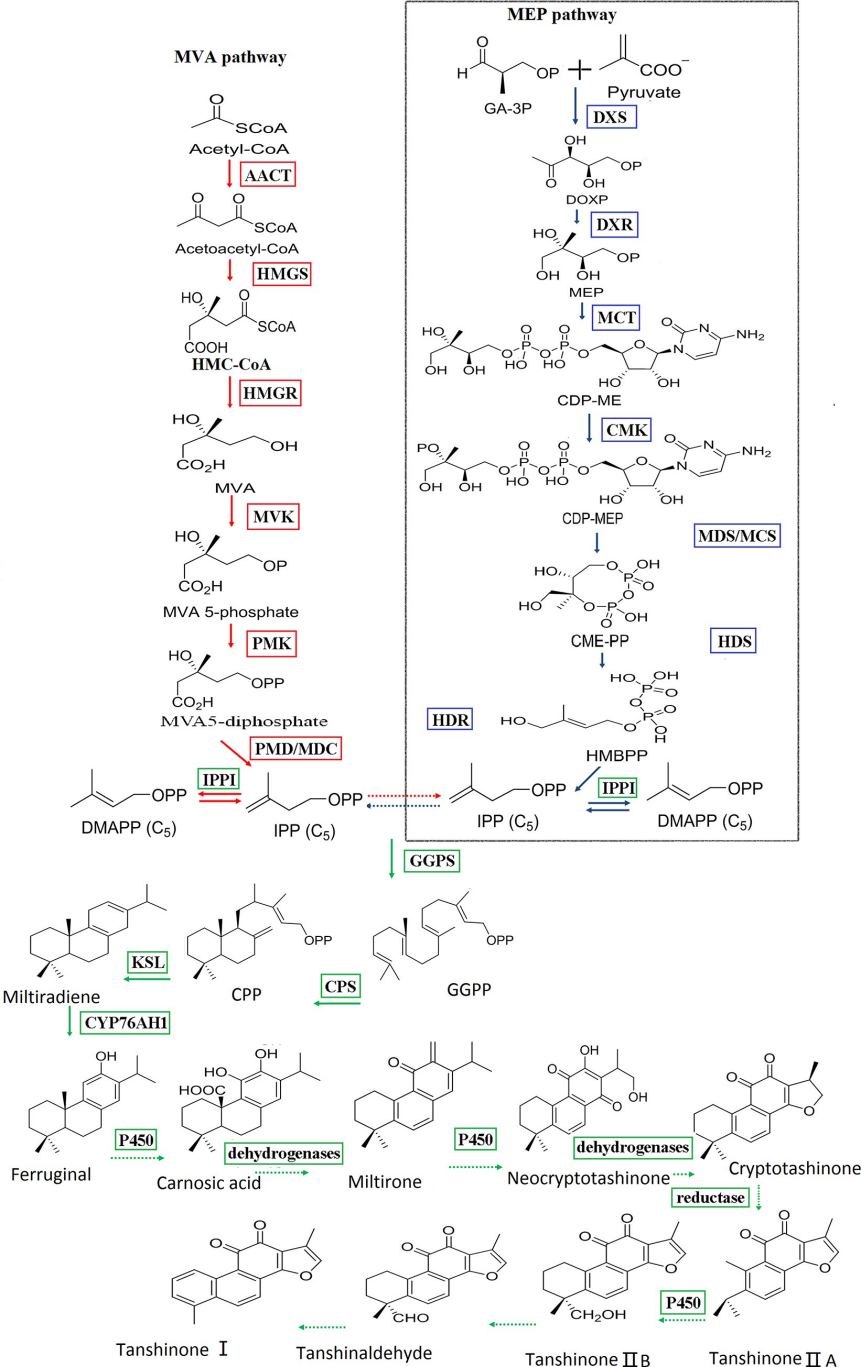
Biosynthesis of tanshinones. A solid arrow represents a known step and a dotted arrow represents an assumed step revised from Ma et al. and Gao et al [8,13]. Enzymes of the MEP pathway (2-C-methyl-D-erythritol 4-phosphate) are as follows: DXS, 1-deoxy-D-xylulose-5-phosphate synthase; DXR, 1-deoxy-D-xylulose-5-phosphate reductoisomerase; MCT, 2-C-methyl-D-erythritol 4-phosphate cytidylyltransferase; CMK, 4-diphosphocytidyl-2-Cmethyl-D-erythritol kinase; MDS, 2-Cmethyl- D-erythritol 2,4-cyclodiphosphate synthase; HDS, 4-hydroxy-3-methylbut-2-enyl diphosphate synthase; and HDR, 4-hydroxy-3- methylbut-2-enyl diphosphate reductase. Enzymes of the mevalonate (MVA) pathway are as follows: AACT, acetyl-CoA acetyltransferase; HMGS, 3-hydroxy-3-methylglutaryl-CoA synthase; HMGR, 3-hydroxy-3-methylglutaryl-CoA reductase; MVK, mevalonate kinase; PMK, 5-phosphomevalonate kinase; and PMD, 5-diphosphomevalonate decarboxylase. Isopentenyl diphosphate isomerase (IPPI) catalyses the isomerisation of dimethylallyl diphosphate (DMAPP) into isopentenyl diphosphate (IPP), whereas conversion of IPP to geranylgeranyl diphosphate (GGPP) is catalysed by geranylgeranyl diphosphate synthase (GGPPS). Copalyl diphosphate synthase (CPS), kaurene synthase-like (KSL) genes and the tanshinone biosynthesis pathway was inferred from previous studies identifying natural diterpenoids from *S*.*miltiorrhiza* [2,14]. These steps include a series of hydroxylation, dehydrogenation, and reduction reactions catalysed by cytochrome P450s, dehydrogenase, and reductase.

*Salvia miltiorrhiza* is one of the most studied medicinal plants and known as a model medicinal plant. It is an important Chinese herbal medicine widely used for treating coronary heart diseases, especially myocardial infarction and angina [1,2]. The active components of *S*. *miltiorrhiza* include both hydrophilic and lipophilic components [3,4]. The lipophilic diterpenoids are generally known as tanshinone compounds, which include tanshinone IIA (TS IIA), tanshinone I (TS I), and cryptotanshinone (CTS), with tanshinone IIA regarded as the most important bioactive ingredient. To date, more than 30 tanshinones and related diterpenoid quinines have been separated and identified [5]. All these diterpenoids have abietic skeleton [6,7]. Tanshinones are particularly complex because of the presence of multiple rate-limiting enzymes [8] that play vital roles in the growth, development, metabolism of the plant, as well as their defence against natural enemies, pathogens, and other competitors [9,10]. Two common precursors of plant terpenoids, including tanshinones, are isopentenyl diphosphate (IPP) and its isomer, dimethylallyl diphosphate (DMAPP), which are synthesised in separate cellular compartments through the mevalonate (MVA) and 2-C-methyl-D-erythritol 4-phosphate (MEP) pathways. The biosynthesis of plant terpenoids can be divided into three stages (Fig 1). At the first stages, IPP and DMAPP are synthesised by the MEP and MVA pathways. 3-Hydroxy-3-methylglutaryl-coenzyme A reductase (HMGR) is an important enzyme in the MVA pathway, and 1-deoxy-D-xylulose 5-phosphate reductoisomerase (DXR) is a key enzyme in the MEP pathway. At the second stages, the intermediate diphosphate precursors, which are important in the synthesis of tanshinones, are synthesised through catalysis by isoprenyl diphosphate synthases, including geranyl diphosphate (GPP), geranyl diphosphate synthase (GPPS), farnesyl diphosphate (FPP), farnesyl diphosphate synthase (FPPS), and geranylgeranyl diphosphate (GGPP) and geranylgeranyl diphosphate synthase (GGPPS). Next, IPP and DMAPP are condensed to form GGPP via catalysis by GGPPS. At the last stages, terpene synthetase/hydrolase (TPSs) catalyses the formation of different terpenoids, such as diphosphate synthetase (CPS), ketal synthetase, and various terpene-modifying enzymes. To date, kaurene synthase-like (KSL) and CPS genes are considered the most important downstream genes in tanshinone biosynthesis [11–14].

It was confirmed that the enzymes involved in terpenoid biosynthesis from the MEP and MVA pathways are located at different subcellular sites. All MEP enzymes are in located in plastids, whereas MVA enzymes are located in the cytoplasm or peroxisome [15–17]. Many genes that encoded key enzymes in terpenoid biosynthesis have been isolated and identified in different plant species [18], such as *Taxus chinensis* [19], *Arabidopsis thaliana* [20,21], and *Hevea brasilien*sis [22,23]. So far, 13 genes that participate in tanshinone biosynthesis have been cloned from *S*. *miltiorrhiza* [8,24–26]. Recently, approximately 56,774 unigenes have been obtained from *S*. *miltiorrhiza* across its entire growth cycle by using Illumina deep sequencing [27]. Moreover, 40 terpenoid biosynthesis-related to genes that encode all the enzymes involved in IPP and DMAPP biosynthesis have been identified [8].

Medicinal plants produce a series of natural and secondary metabolites when they grew and adapted to the environment during their survival. Thus, certain biological and abiotic factors can stimulate, induce, or influence the production of these secondary metabolites; for example, tanshinone production in the roots of *S*. *miltiorrhiza* can be induced by biotic (carbohydrate part of yeast extract) and abiotic inducers (Ag^+^ and Cd^2+^) [28–33] or phytohormones (salicylic acid and methyl jasmonate) [12,34,35]. It is generally believed that large amounts of chemical fertilisers reduce herb quality. Soils with relatively poor nutrient level are conducive for the synthesis of secondary metabolites of medicinal plants. Among these nutrients, N plays an important role in the synthesis of secondary metabolites. It is well known that the soil where wild medicinal plants grow is very nutrient poor, but this condition enhances the accumulation of beneficial medicinal components. Many studies have shown that low-nitrogen stress promotes the production of secondary metabolites such as ketones, terpenoids, and phenolic substances [36–40]. Moreover, N also influenced the synthesis and metabolisms of lipid and starch [41–42]. For example, under nitrogen starvation stress, the levels of both starch and lipid in *Chlorella zofingiensis* initially increased, but the starch level partially decreased after 2 days of N stress [43]. Furthermore, nitrogen stress is key to ensure the quality of lipids; for example, algal cells grown in no-nitrogen medium (N stress) had neutral lipid content of 86.7% of total lipids [44].

Nitrogen is the main component of proteins and it is involved in plant growth and developmental processes, including photosynthesis [45], seed dormancy [46], root growth [47], and flowering time [48]. In addition, N is a major nutritional factor that can improve the yield of crop plants. However, excessive use of a nitrogen fertiliser inhibits plant growth and development. Hence, decreasing the use of nitrate fertiliser without affecting yield and product quality is one of the aims of precision agriculture.

Many studies have shown that low-nitrogen stress affects the expression of genes and signal molecules related to plant secondary metabolites, and that microRNAs indirectly or directly regulate the transcription of genes related to the synthesis of secondary metabolites [49–51]. Regulatory miRNAs may promote plant adaptation to low-nitrogen stress by regulating hormone signal transduction, thus providing valuable clues for investigating the regulation of hormone signal transduction by miRNA under low-nitrogen stress [52].

In our previous study, tanshinone concentration in the roots of *S*. *miltiorrhiza* were relatively higher under low-N stress than under normal-N supply [53]. However, the molecular mechanism of low N stress on tanshinone biosynthesis is still unclear. In this study, we investigated the expression profiles of the genes involved in the biosynthesis of tanshinones in *S*.*miltiorrhiza* under nitrogen-free, low-nitrogen, and normal-nitrogen conditions using transcriptome (RNA-seq) technology. We also analyzed the expression of key enzymes changes in the biosynthesis pathway and molecular regulation of tanshinones in *S*. *miltiorrhiza* roots under nitrogen-free and low-nitrogen conditions. Furthermore, quantitative analysis of tanshinones (including TS I, TS IIA, and CTS) was also conducted by liquid chromatography analysis. The results of this study would reveal the mechanism of low nitrogen stress on tanshinone synthesis of *Salvia miltiorrhiza*, this study would provide a theoretical basis for further study on the molecular mechanism and genetic regulation of secondary metabolite biosynthesis under nitrogen fertilizer in *S*. *miltiorrhiza*.

## Materials and methods

### Plant material

*S*. *miltiorrhiza* seedlings (2 months old; approximately 8–10 cm tall) were transplanted in soilless quartz sand as a matrix in a greenhouse at the Institute of Medicinal Plant Development. The plants were watered every fifth day with nutrient solution containing 4 N levels: 0 mmol·L^-1^ N (N-free (N0)), 4 mmol/L N (2 mmol/L Ca(NO_3_)_2_·4H_2_O) (low-N (Nl)), 8 mmol/L N (4 mmol/L Ca(NO_3_)_2_·4H_2_O) (middle-N (Nm)), and 16 mmol/L N (8 mmol/L Ca(NO_3_)_2_·4H_2_O) (full-N (Nf, as control)). The detailed method has been described in our previous report [53], these four nitrogen treatments simultaneously contain other nutrient solutions such as 2.5 mmol/L K_2_SO_4_, 2 mmol/L MgSO_4_·7H_2_O, 1 mmol/L KH_2_PO_4_, 0.045 mmol/L H_3_BO_3_, 0.01 mmol/L MnCl_2_·4H_2_O, 0.8 μmol/L ZnSO_4_·7H_2_O, 0.3 μmol/L CuSO_4_·5H_2_O, 0.4 μmol/L Na_2_MoO_4_·2H_2_O, 0.02 μmol/L FeSO_4_·7H_2_O, and 0.02 μmol/L EDTA-Na_2_ (pH = 6). At 30, 45, 60, 75, and 90 DAT, the N-treated (low, medium, and normal-N levels) and non-N-treated roots were collected. Each sample was divided into two parts, one of which was immediately frozen in liquid nitrogen and stored at -80°C for total RNA isolation and metabolome analysis, and the other was dried quickly with an absorbent paper for investigation of tanshinone content. The roots with similar thickness and size were selected for transcriptome, metabolome, and chemical analyses.

### RNA isolation, library preparation, and sequencing

Plant RNeasy kit (Bio-Teke, Beijing, China) was used to isolate total RNA from the roots of *S*. *miltiorrhiza* grown under N0, Nl, and Nf conditions at 45, 60, and 75 DAT. Next, EtBr-stained 1% agarose gels were used to monitor RNA contamination and degradation, and a spectrophotometer of NanoPhotometer® (IMPLEN, Westlake Village, CA) was used to analyse the purity of the RNA. RNA Assay Kit Qubit® (2.0) Fluorometer (Life Technologies, Foster City, CA) was used for assessing RNA concentration, Nano-6000 Assay Kit (Bioanalyzer 2100; Agilent Technologies, Santa Clara, CA) was used to assess RNA integrity. An cDNA sample preparation Kit (Illumina TruSeq™, San Diego, CA) was used to construct sequencing libraries. Each measurement was performed with two biological replicates. Therefore, 18 libraries were constructed and sequenced with the paired-end method using an Illumina instrument (Hiseq^TM^ 2000, USA).

### Quality assessment and *de Novo* assembly

NGS QC Toolkit was used to process raw data [54]. Clean reads were obtained by rejecting ploy-N-containing and low-quality reads. We used Trinity (Version: trinityrnaseq_r20131110) for transcript assembly, and TGICL software (http://tophat.cbcb.umd.edu/) was applied to generate effective unigenes with the default parameter values [55,56].

### Differential gene expression and functional annotation and classification

The fragments per kilobase of transcript per million fragments mapped value of each gene was calculated by cufflinks [57,58], and htseq-count was applied to obtain the read counts of each gene [59]. The DESeq function of estimateSizeFactors and nbinomTest was used to analyse DEGs in the nine groups of samples with two biological replicates [60–63]. Gofeat was used to annotate the functions of the genes. Protein function and Gene Ontology (GO) term information was then extracted from the Gofeat report [64]. Functional annotations were performed by BLAST comparisons against the manually controlled KEGG (Genes database) by using KEGG Automatic Annotation Server [65]. HISAT2 (2.0.4) was used for genome alignment [66]. Feature count from subread [67] was then used to extract the read counts for the genes [59] from aligned bam files. Read count matrix was imported into R to identify DEGs using DESeq2 [68]. DEGs were then extracted for GO and KEGG analyses by a cluster profiler [69].

### qPCR validation

An Enzyme Mix I (PrimerScript RT, TaKaRa, Japan) was used for qPCR analysis with a PCR System (GeneAmp^®^ 9700; Applied Biosystems, USA). Total RNA was extracted from *S*. *miltiorrhiza* roots, and the purity and integrity of the RNA was determined by agarose gel electrophoresis and an RNA Assay Kit (Nano 6000, USA) and Bioanalyzer 2100 system (Agilent Technologies, Santa Clara, CA). An RT-PCR Instrument (LightCycler^®^ 480 II; Roche, Swiss) was used for PCR. The PCR reaction mixture (10 μl) consisted of 1 μl of cDNA, 5 μl of 2× SYBR Green I Master (LightCycler^®^ 480, Roche, Swiss), 3.6 μl of nuclease-free water, 0.2 μl of forward primer, and 0.2 μl of reverse primer. The reaction was conducted in a 384-well optical plate (Roche) for 10 min at 95°C, followed by 40 cycles at 95°C for 10 s and at 60°C for 30 s. At the end of the PCR cycle, melting curve analysis was conducted to verify the specific production of an expected PCR product. The primer sequence was designed and synthesised by general biotech (general, PRC, China) according to mRNA sequences from the NCBI database, as shown in S1 Table. The mean value of three replicates was standardised with that of 18S as an internal control. The relative levels of genes were calculated by the 2^-ΔΔCt^ method [70]. Three replicates were used for each sample.

### GC/TOF-MS analysis

Roots with similar thickness or size were selected and crushed in liquid nitrogen and freeze-dried at -50°C for 8 h for GC/TOF-MS analysis. A gas chromatography system (Agilent 7890; Agilent) and a Pegasus HT time-of-flight mass spectrometer were used for GC/TOF-MS analysis. The DB-5MS capillary column was coated with 5% of diphenyl and 95% of dimethylpolysiloxane (film thickness: 0.25 μm; inner diameter: 30 m × 250 μm) (J&W Scientific, Folsom, CA, USA). Next, 1 μl aliquots of analyte was injected into the system at the non-splitting mode. Helium was used as a carrier gas, the purging flow at the front inlet was 3 ml/min, and the column gas flow was 1 ml/min. The initial temperature was maintained for 1 min at 50°C, then increased to 290°C at a rate of 10°C/min, and then maintained for 10 min at 290°C. The temperatures of implantation (injection), transmission line, and ion source were 280°C, 270°C, and 220°C, respectively. The electron collision energy mode was -70 eV. Mass spectrum data were obtained at the range of 50–500 M/Z at the rate of 20 spectra per s after 460 s of solvent delay. The LECO-Chroma TOF4.3X software and a LECO-Fiehn Rtx5 database were applied to extract the original peak, filter and correct the baseline, align the peak, perform deconvolute analysis, identify the peak, and integrate the peak area [71]. The peak was identified by retention time index (RI); the RI tolerance was 5000. Each treatment group was analysed with two technical and five biological replicates.

### Analysis of active components

Among samples from different treatment groups, red roots (≥2mm) were dried, ground, and prepared for analysis. Each treatment group had five biological repetitions. Tanshinones (TS IIA, TS I, and CTS) levels were determined by high-performance liquid chromatography (Waters 1525 system; Waters Technologies, Milford, MA, USA), and the total tanshinone level was determined by ultraviolet spectrophotometry (UV2450; Shimadzu, Japan). The sample analytical condition and preparation have been described by He et al. (2010) and Lu et al. (2015) [56,72]. The roots were too small for analysis on the first test stage, and thus transplanted for 30 days and the content of the four tanshinones was examined only once. Therefore, the data of this stage were not included in further statistical analyses.

### Nitrate reductase and glutamine synthetase analysis

Nitrate reductase determination: Weigh 0.300 g root samples, add 0.1211 g cysteine, 0.0372 g EDTA extraction buffer into 10 mL centrifuge tube, then grind on ice to form a homogenate, and centrifuge at 12000 r.min^-1^ and 4℃ for 15 min, the supernatant which is the crude enzyme extract. and then was determined by ultraviolet spectrophotometry (UV2450; Shimadzu, Japan) (520 nm) by using aminobenzenesulfonic acid-naphthylamine colorimetric method.

Glutamine synthetase determination:Weigh 0.300g root samples, add phosphate buffer (containing 0.5 mmol.L^-1^ EDTA, 50 mmol.L^-1^ K_2_SO_4_, pH 7.2) into 10 mL centrifuge tube and grind on ice to form a homogenate, The supernatant obtained after centrifugation at 12000 r.min^-1^ and 4 ℃ for 20 min, which is the crude enzyme extract. Add 1ml color developer (TCA, FeCl_3_, HCl mixed color developer) into the crude enzyme extract, and was determined by ultraviolet spectrophotometry (UV2450; Shimadzu, Japan) (540 nm).

### Statistical analysis

Duncan multipass test and student’s *t*-test were conducted to examine the data of physiological characters and gene expression by using a statistical software (SPSS 15.0.1(IBM, USA); microsoft-2013 (USA). Differences with *P* < 0.05 and *P* < 0.01 were considered significant and highly significant, respectively.

## Results

### Effects of N starvation on the biomass of *S*. *miltiorrhiza* roots

**Fig 2:**
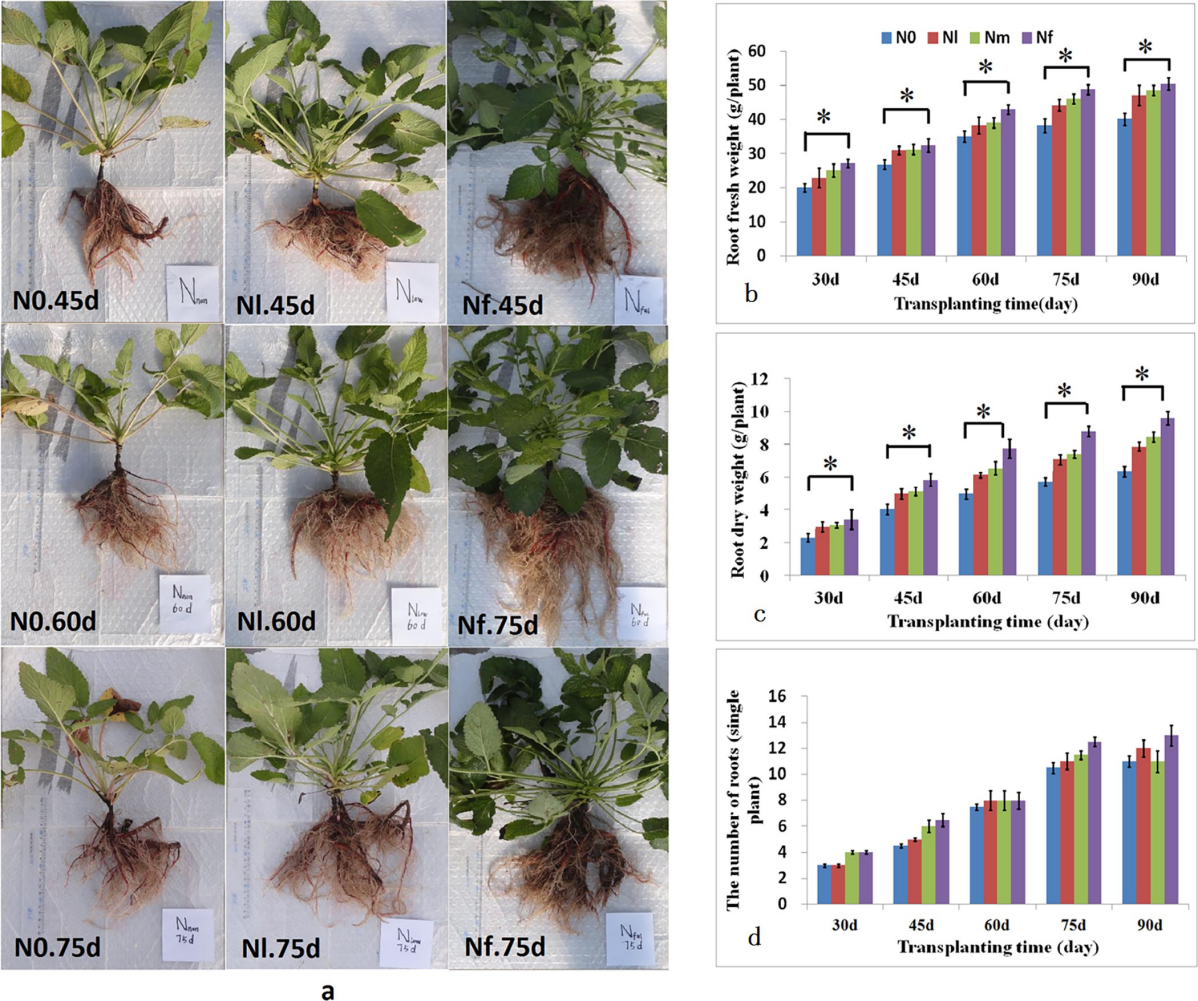
The effects of low nitrogen level on the growth in *S*. *miltiorrhiza* roots at 45, 60, 75, and 90 days after transplanting (DAT). (a) Phenotypes of *S*. *miltiorrhiza* under Full-N (Nf) and N-deficient conditions (Non-N(N0) and Low-N (Nl)) at the indicated times (Photographed by Li-Lan Lu). (b) Fresh weight of the roots under full-nitrogen (Nf), middle level-nitrogen (Nm), low-nitrogen (Nl), and non-N (N0) conditions at the indicated times. (c) Dry weight of the roots, (d) number of the roots. Values are presented as means ± SD, n = 5. Asterisks (*) indicate significant differences *(P* < 0.05, Student’s *t*-test).

To elucidate the effects of different N concentrations on the root growth in *S*. *miltiorrhiza*, both the fresh and dry weight of the roots were examined after growth under nitrogen-free (N0), low-nitrogen (Nl), medium-nitrogen (Nm), and full-nitrogen (Nf) conditions. Root dry weights showed a stable increasing trend during the five test periods (30, 45, 60, 75, and 90 days after transplanting (DAT)) (Fig 2B and 2C). There was no obvious difference in root dry weights between Nl and Nm during all test stages. The fresh and dry weight of roots of *S*. *miltiorrhiza* under Nf treatment were significantly higher than those under N0 treatment; however, there was no significant difference in the fresh and dry weight between the Nf, Nm, and Nl groups during the five test periods. Moreover, there was no significant difference in the number of roots (diameter > 2 mm) of *S*. *miltiorrhiza* among the N0, Nl, Nm, and Nf groups (Fig 2C). In addition, the root colour of *S*. *miltiorrhiza* (red or brown red) was observed and identified qualitatively. The colour of the roots grown under low-nitrogen levels (N0 and Nl) was deeper than that of those grown under normal-nitrogen level (Nf) (Fig 2A).

### Effects of N starvation on the accumulation of tanshinones in *S*. *miltiorrhiza* roots

**Fig 3:**
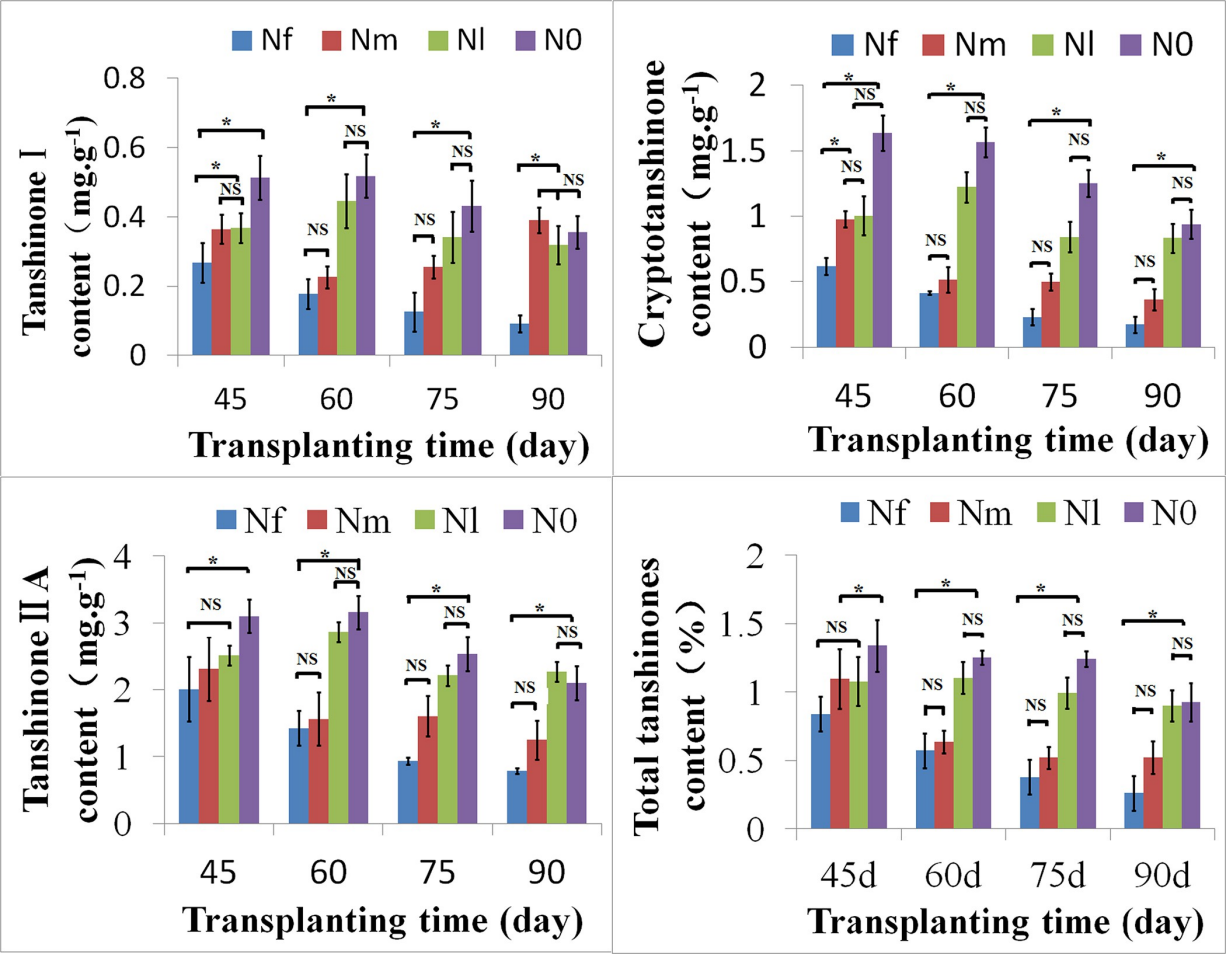
Low nitrogen influencing on the tanshinones in roots of *S*. *miltiorrhiza* at 45, 60, 75, and 90 days after transplanting (DAT), respectively. Values are as means ±SD, n = 5. Asterisks represented significant differences between treatments, NS indicate no significant differences (Student’s *t*-test, P < 0.05).

To examine the effect of different N conditions on the accumulation of tanshinones, the levels of TS IIA, TS I, CTS, and total tanshinones (TTS) were investigated at 45, 60, 75, and 90 DAT under N0, Nl, Nm, and Nf treatments. The results showed that the levels of these four compounds in *S*. *miltiorrhiza* roots under Nl and N0 treatments were significantly higher than that under Nf treatment at 45, 60, 75, and 90 DAT (Fig 3). For example, TS I contents under the N0 and Nl treatments were 3.4- and 2.7-fold higher, respectively, than that under the Nf treatment on day 75 (0.432 vs. 0.126 mg/g and 0.342 vs. 0.126 mg/g (dry weight)). There were no obvious differences in the TS IIA, TS I, CTS, and TTS contents between the Nm and Nf treatments. Moreover, under Nm and Nf conditions, the content of the four tanshinones showed a decreasing pattern during the tested stages, with the exception of TS I content under the Nm treatment. Furthermore, there was almost no significant difference in the content of the four tanshinones between the third (75 DAT) and last stage (90 DAT) under the four treatments. Therefore, we conducted transcriptome sequencing and metabolite analysis on the roots of *S*. *miltiorrhiza* under N0, Nl, and Nf treatments and at 45, 60, and 75 DAT, respectively.

### mRNA sequencing, assembly

To study mRNA expression in *S*. *miltiorrhiza* under low-N stress, mRNA-seq libraries were constructed and sequenced from *S*. *miltiorrhiza* cultivated at N0, Nl and Nf treatments. From the groups treated with N0, Nl, and Nf, 70316750, 70423240, and 70362023 average values of clean paired-end reads were generated, respectively. The Q30 percentages of the three mRNA-seq libraries was not less than 93.10% (S2 Table). The total length of the unigenes was 138,424 bp, which ranged at 201–28,091 bp. The length of N50 was 2,197 bp, and the average length was 1296.57 bp (S3 Table, S1 File). In general, the combination integrity increased when the N50s of the unigenes were at ≥800 bp. This combination produced many unique sequences of different lengths: 44,442 (32.11%) with a length of 200–500 bp, 35,861 (25.91%) with a length of 500–1,000 bp, 28,088 (20.29%) with a length of 1,000–2,000 bp, and 30,033 (21.70%) with a length of > 2,000 bp (S1 File). These results showed that most of the unique sequences were longer than 500 bp (93982, accounting for 67.89%), thus providing sufficient sequence length to allow accurate annotation.

### Identification of differentially expressed genes (DEGs)

**Fig 4:**
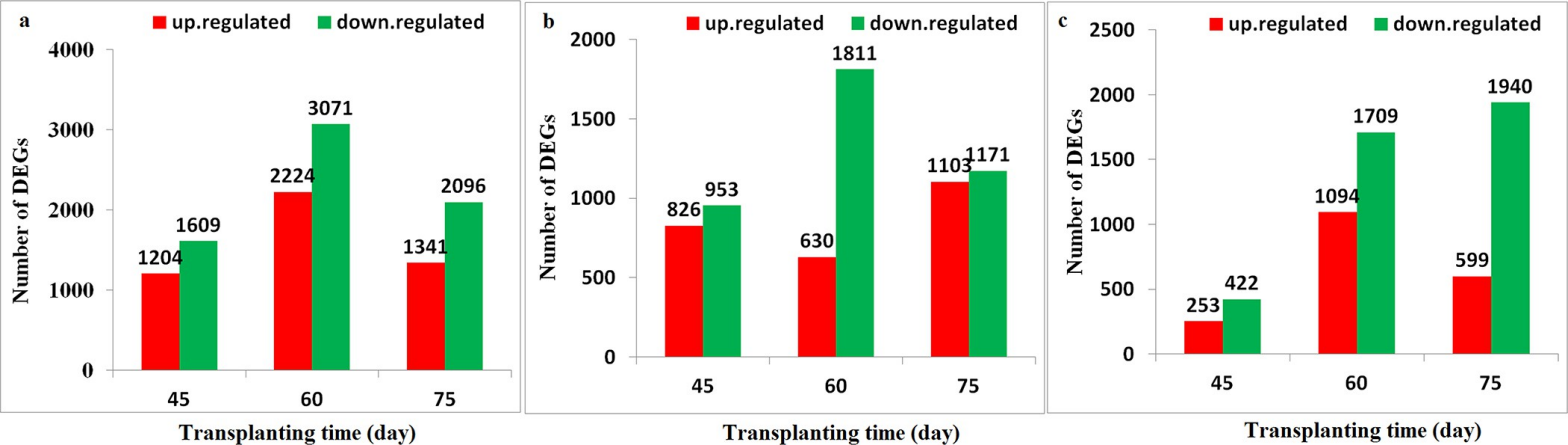
Number and distribution of up- and downregulated genes in the roots of *S*. *miltiorrhiza* after treatment with different N levels at 45, 60, and 75 days after transplanting (DAT). (a) N0 vs. Nf. (b) Nl vs. Nf. (c) N0 vs.Nl.

**Fig 5:**
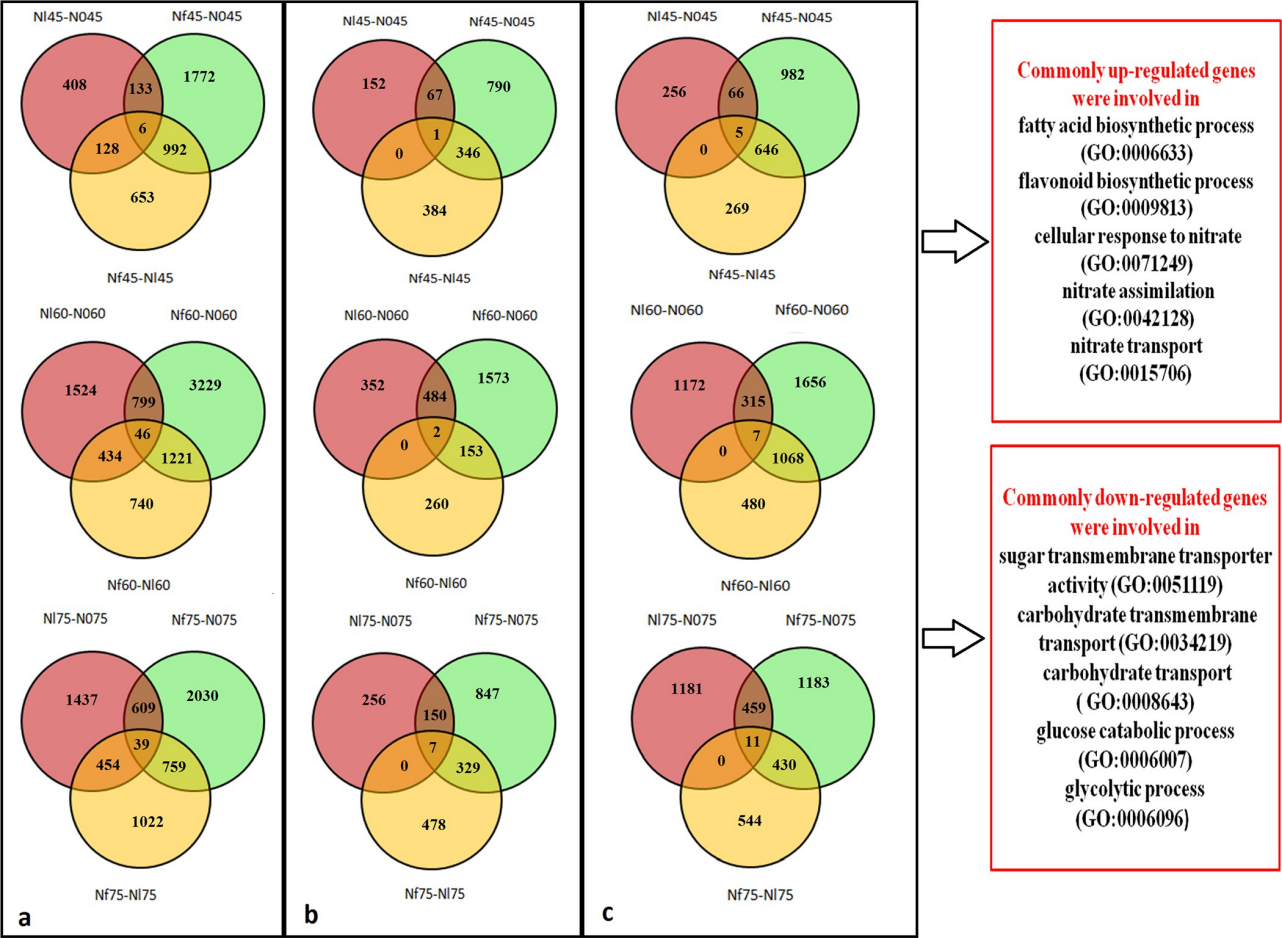
Venn diagram of the number of genes in the Nf-N0 (N0 compared to Nf), Nf-Nl, and Nl-N0 groups at 45, 60, and 75 days after transplanting (DAT). (a) All differentially expressed genes (DEGs), (b) upregulated DEGs, (c) downregulated DEGs.

To obtain an overall view the effect of low-N condition on metabolite production during the development and growth of *S*. *miltiorrhiza*, the expression profiles of genes in the roots under N0, Nl, and Nf conditions were analysed. We used a strict value of FDR < 0.01 and log2fc ≥ 1 as thresholds to judge the significance of differences in gene expression. At all three developmental stages, the number of DEGs (up- and downregulated genes) was different and the number of upregulated genes was less than that of downregulated genes. For example, a total of 3,437 (1,341 upregulated and 2,096 downregulated), 2,274 (1,103 upregulated and 1,171 downregulated), and 2,539 (599 upregulated and 1,940 downregulated) genes were differently expressed in N0 vs. Nf, Nl vs. Nf, and N0 vs. Nl at 75 DAT (Fig 4C). At the same treatment time, the number of DEGs (up- and downregulated genes) was the lowest in Nl (S1 Fig). Various genes involved in growth and developmental processes responded to N-free condition, especially at 60 DAT, when flowering occurred. Furthermore, in the early period (45 DAT). In addition, there were 922, 1,221, and 759 common genes between N0 vs. Nf and Nl vs. Nf, respectively, across 45, 60, and 75 DAT, which were the greatest among the compared groups (N0 vs. Nf, Nl vs. Nf and N0 vs. Nl). Differential expression analysis showed among N0 vs. Nf, Nl vs. Nf, and N0 vs. Nl, respectively, across 45, 60, and 75 DAT were identified 6, 46, and 39 common genes; 1, 2, and 7 commonly upregulated genes; and 5, 7, and 11 commonly downregulated genes, (Fig 5). The commonly upregulated genes belong to the fatty acid biosynthetic process (GO:0006633), the flavonoid biosynthetic process (GO:0009813), cellular response to nitrate (GO:0071249), nitrate assimilation (GO:0042128), and nitrate transport (GO:0015706), whereas the commonly downregulated genes are involved in sugar transmembrane transporter activity (GO:0051119), carbohydrate transmembrane transport (GO:0034219), carbohydrate transport (GO:0008643), glucose catabolic process (GO:0006007), and the glycolytic process (GO:0006096) (S4 and S5 Tables).

### GO pathway enrichment analysis of *S*. *miltiorrhiza* in response to N starvation stress

**Fig 6:**
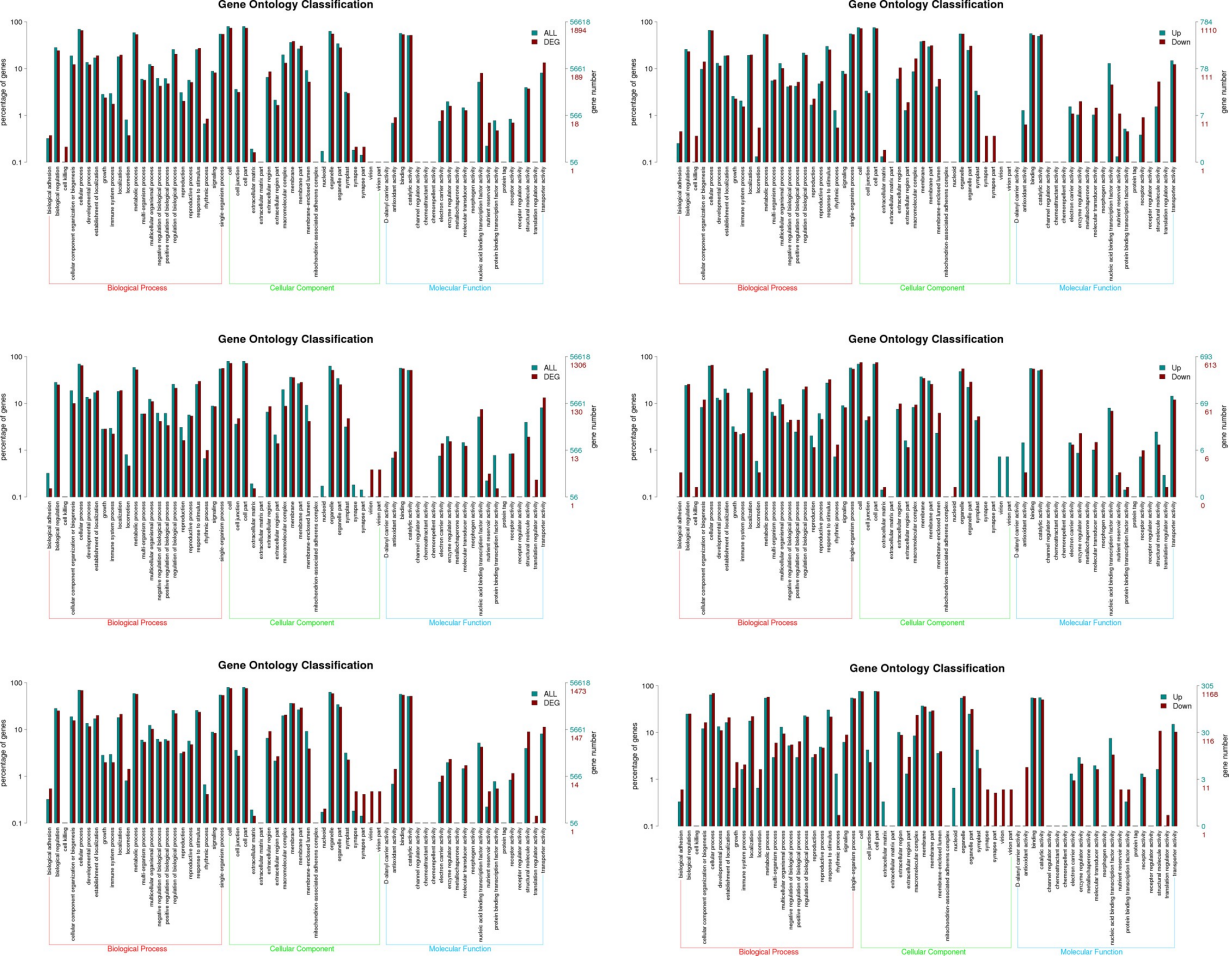
Histogram of GO classification of differentially expressed genes (DEGs) in N0 vs. Nf, Nl vs. Nf, and N0 vs. Nl at 75 days after transplanting (DAT). Blue represents all unigenes annotated in each subcategory, red represents all DEGs annotated in each subcategory. The right y-axis represents the number of genes annotated in each subcategory. The left y-axis represents the percentage of unigenes annotated or DEGs in the main category. (a, b) N0 vs. Nf, (c, d) Nl vs. Nf, (e, f) N0 vs. Nf.

We performed GO enrichment analysis with the DGEs and described their function by combining the GO annotation results of N0 vs. Nf and Nl vs. Nf at 45, 60, and 75 DAT, respectively. With a BLASTX hit using Blast2 GO (*P*<0.05), GO categories were assigned with more than 700 DEGs between N0 vs. Nf and Nl vs. Nf at 45, 60, and 75 DAT, respectively (S6 Table). The DEGs and up- and downregulated genes in the GO distribution function among N0 vs. Nf and Nl vs. Nf were very similar. The genes associated with catalytic activity (GO: 0003824) (~50%), binding (GO: 0005488) (~50%) (molecular function), cell (GO: 0005623) (~70%), cell part (GO: 0044464) (~70%), organelle (GO: 0043226) (~50%) and membrane (GO:0016020) (~40%) (cellular component), metabolic process (GO: 0008152) (~50%), cellular process (GO: 0009987) (~60%), single-organism process (GO: 0044699) (~50%), and response to stimulus (GO: 0050896) (~30%) (biological process) were significantly enriched (Figs 6 and S2). Most of these GO terms were consistent with those in previous studies [12,27], showing the validity of our data set. Details of the topGO enrichment of these groups are shown in S3 Fig and S7 Table.

The topGO enrichments showed that the biological processes “phosphate ion transmembrane transport” and “phosphate ion transport” were the most abundant functional groups enriched by downregulated DEGs in N0 vs. Nf at 45 and 75 DAT as well as in Nl vs. Nf at 45 DAT. The terms “response to chitin” and “phosphate ion transport” were obviously enriched by downregulated DEGs in N0 vs. Nf at 60 DAT, and at the same time, the terms “response to chitin”, “response to wounding”, “phosphate ion transport”, and “phosphate ion transmembrane transport” were enriched in Nl vs. Nf by downregulated DEGs. In addition, at 75 DAT, the process “response to water deprivation” was enriched significantly by downregulated DEGs in Nl vs. Nf. Moreover, wounding and deprivation processes were downregulated under Nl treatment at 60 and 75 DAT, Furthermore, “glucose−6−phosphate transport” and “phosphoenolpyruvate transport” were enriched by upregulated DEGs under N0 treatment at 45 DAT. The term of “transcription, DNA-templated” was markedly enriched by upregulated DEGs under N0 treatment at 60 and 75 DAT. The processes of cholesterol metabolic, lipid catabolism, and nucleoside or amino acid transport were enriched significantly by upregulated DEGs under Nl condition at 45, 60, and 75 DAT (S3 Fig, S7 Table).

The cellular component “integral component of plasma membrane” was enriched by upregulated DEGs in N0 vs Nf at 45 DAT; nevertheless, “chloroplast” and “central vacuole” were enriched by downregulated DEGs. However, these two terms, “plant-type vacuole membrane” and “integral component of plasma membrane”, were also enriched in Nl vs. Nf at 45 DAT. Similarly, the terms “integral component of membrane” and “plasma membrane” were enriched in N0 vs. Nf and Nl vs. Nf at 60 DAT. The term “integral component of plasma membrane” was also enriched in N0 vs Nf at 60 and 75 DAT, respectively. Moreover, “plant−type vacuole membrane” was enriched in N0 vs. Nf and Nl vs. Nf at 75 DAT (S3 Fig, S7 Table).

Under the molecular function category, “phosphate ion transmembrane transporter activity” was enriched by downregulated DEGs in N0 vs. Nf and Nl vs. Nf in almost all treatments and stages except in Nl treatment at 75 DAT. Moreover, “transcription factor activity” and “sequence-specific DNA binding” were obviously enriched by upregulated DEGs in N0 vs. Nf at 60 and 75 DAT. At the same time, “mannose binding” and “mRNA methyl transferase activity” were also enriched by upregulated DEGs in Nl vs. Nf at 60 DAT. Further, 1-deoxy-D-xylulose-5-phosphate reductoisomerase (DXR) was remarkably enriched by upregulated DEGs under N0 and Nl conditions at 45 or 60 DAT. The DEGs encoding the enzyme 4-hydroxy-3-methybut-2-en-1-yl diphosphate synthase (HDS) were upregulated under N0 treatment at 75 DAT (S3 Fig, S7 Table).

### Kyoto Encyclopaedia of Genes and Genomes (KEGG) pathway enrichment analysis of *S*. *miltiorrhiza* in response to N starvation stress

**Fig 7:**
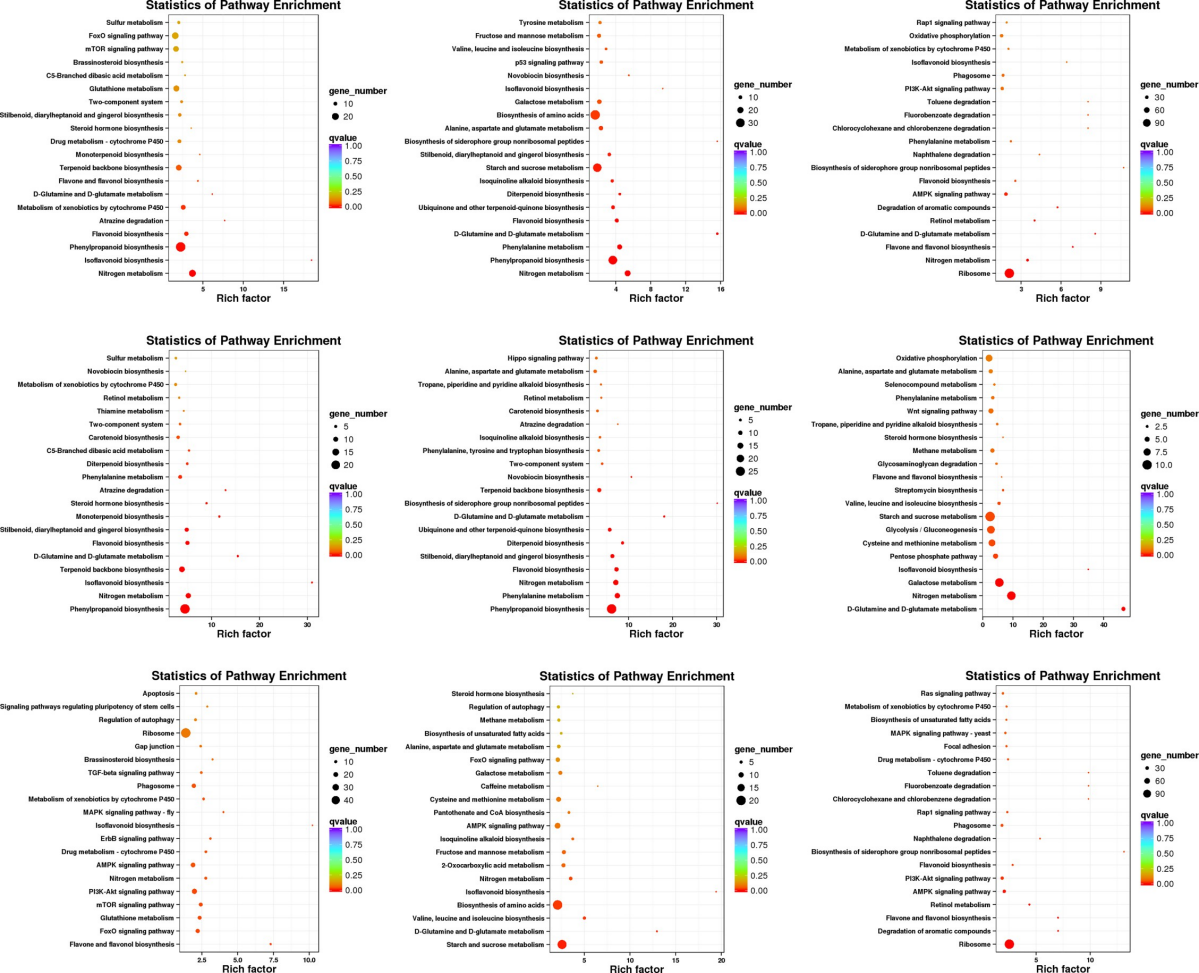
KEGG enrichment pathways in N0 vs. Nf, Nl vs. Nf, and N0 vs. Nl at 75 days after transplanting (DAT). All differentially expressed genes, including upregulated and downregulated DEGs, were distributed in the KEGG pathways. The considerably changed pathways indicate the most abundant dots. The rich factor was the ratio of the number of DEGs in a pathway and the number of all genes involved in this pathway. The degree of gene enrichment was enhanced by increasing rich factor and decreasing (Q-value). The Q-value was the rectified by P-value (FDR). (a) All DEGs in N0 vs. Nf, (b) all DEGs in Nl vs. Nf, (c) all DEGS in N0 vs. Nl, (d) upregulated DEGs in N0 vs. Nf, (e) upregulated DEGs in Nl vs. Nf, (f) upregulated DEGs in N0 vs. Nl, (g) downregulated DEGs in N0 vs. Nf, (h) downregulated DEGs in Nl vs. Nf, (i) downregulated DEGs in N0 vs. Nl.

There were more than 100 differentially expressed pathways under N0 and Nl conditions at 45, 60, and 75 DAT (S8 Table). In this study, we used enrichment factor and Q-value to analyse the significance of the pathways. The results of the first 20 minimum Q-value pathways are shown in Fig 7. The dominant and significantly enrichment pathways are shown in S8 Table. The enrichment analysis of all DEGs annotated from the KEGG pathways revealed that all DEGs involved in photosynthesis, nitrogen metabolism, galactose metabolism, glycerolipid metabolism, and phenylpropanoid biosynthesis were significantly enriched by N starvation (N0 and/or Nl) at 45, 60, and 75 DAT. Under N0 condition, terpenoid backbone biosynthesis was enriched by upregulated DEGs at all three stages, and similar results were also obtained under Nl condition at 45 DAT. In addition, at 60 DAT, plant hormone signal transduction and phenylpropanoid biosynthesis were enriched by upregulated DEGs under N0 treatment. However, both nitrogen metabolism and photosynthesis pathways were enriched by downregulated DEGs under N0 or Nl conditions at the early period (45 DAT), and flavone and flavonol biosynthesis was enriched by downregulated DEGs under N0 condition at 75 DAT. At the same time, downregulated DEGs enriched starch and sucrose metabolism pathways. Through KEGG mapping, N starvation was found to mainly affect the MEP pathway at 45 and 75 DAT. Few DEGs were mapped under Nl condition compared to those under Nf at 60 DAT, but three DEGs encoding enzymes of the MVA pathway were downregulated under Nl condition (S4 Fig, S9 and S10 Tables).

### Nitrogen-related enzyme activity and genes in response to N starvation stress

**Fig 8:**
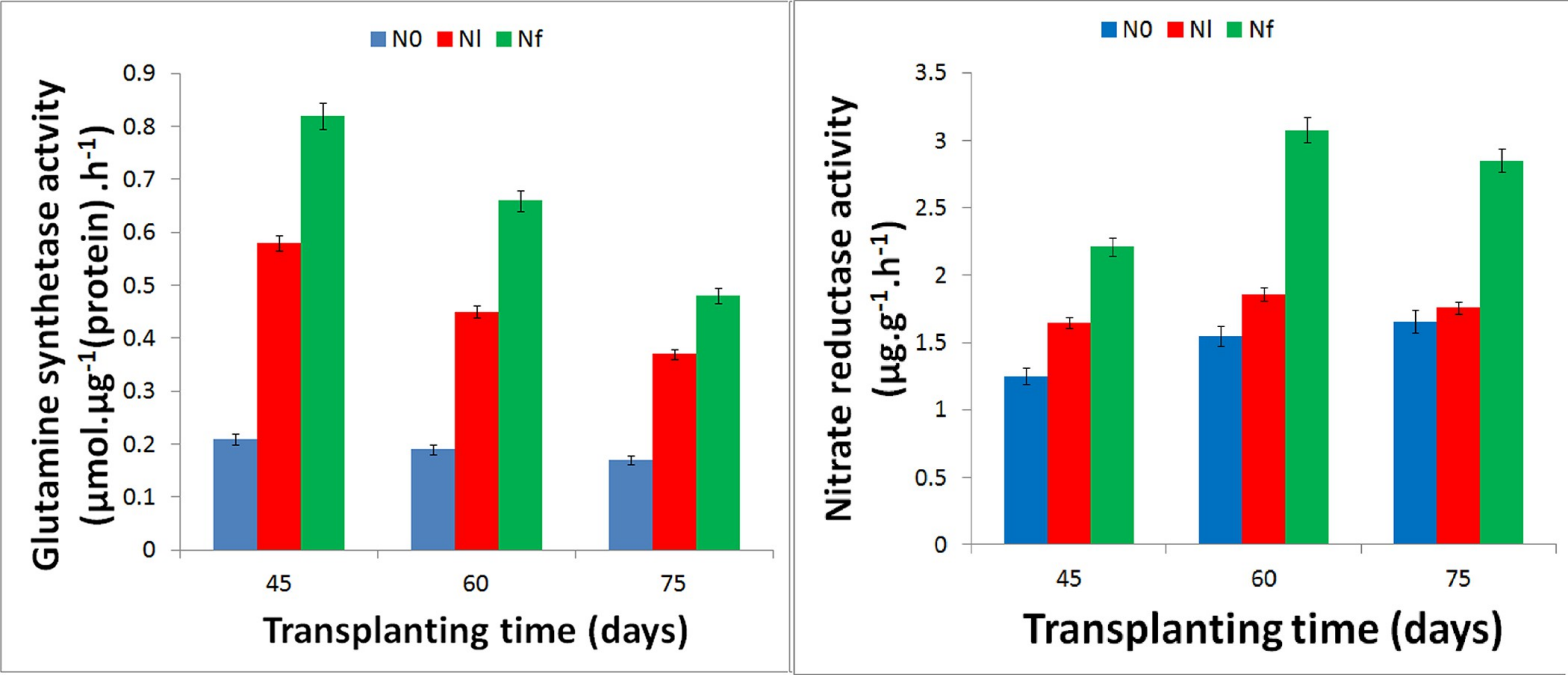
Activities of glutamine synthetase (GS) and nitrate reductase (NR) in *S*. *miltiorrhiza* roots under normal nitrogen supply and nitrogen starvation.

**Fig 9:**
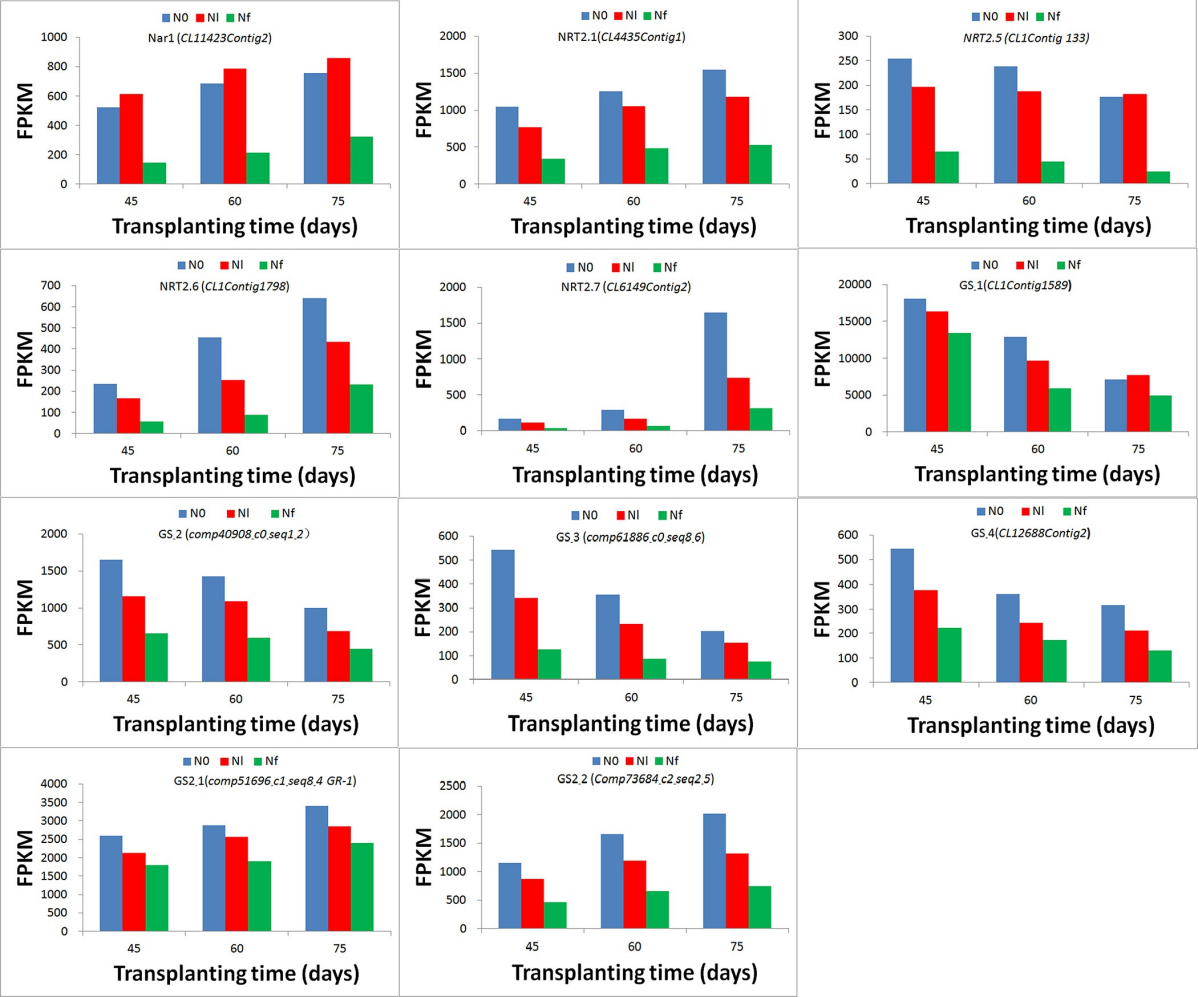
Transcription expressions (FPKM) of GS family genes (GS1, GS2), Nar1 and nitrate transporter genes NRT2 family (NRT2.1, NRT2.5, NRT2.6, NRT2.7) in *S*. *miltiorrhiza* roots under normal nitrogen supply and nitrogen Starvation.

The Fig 8 showed that the nitrate reductase activity in the roots under nitrogen starvation is reduced, while the glutamine synthetase activity is increased, compared with the normal nitrogen supply. Compared with the normal nitrogen supply, under nitrogen starvation treatment, the transcription (FPKM) expressions of GS family genes (GS1, GS2), Nar1 and nitrate transporter genes NRT2 family (NRT2.1, NRT2.5, NRT2.6, NRT2.7) in roots, was significantly up-regulated (P<0.01) (Fig 9).

### DEGs of tanshinone biosynthesis-related genes in response to N starvation stress

**Fig 10:**
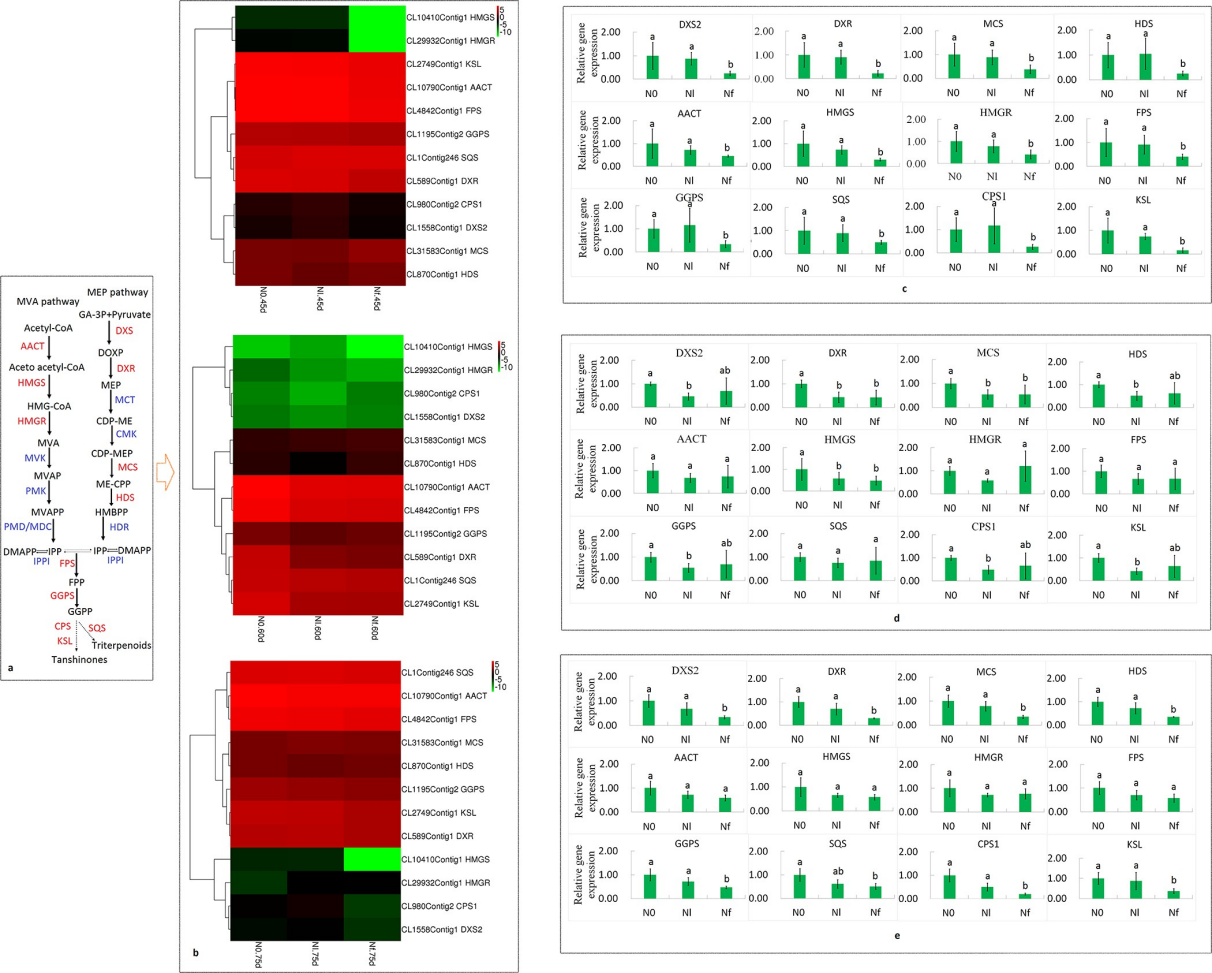
Proposed terpenoid biosynthesis pathways in *S*. *miltiorrhiza* (a). Heat map describing the expression profile of tanshinone biosynthesis-related genes from the MEP and MVA pathways in the roots of *S*. *miltiorrhiza* grown under low and full-nitrogen levels in N0, Nl, and Nf at 45, 60, and 75 days after transplanting (DAT) (b). qRT-PCR validation of differentially expressed genes in N0, Nl, and Nf at 45 (c), 60 (d), and 75 (e) DAT. Different letters represent significant differences between treatments (P < 0.05).

The expression profiles of tanshinone biosynthesis-related genes from the three stages of terpenoid biosynthesis were analysed using the RNA-seq data (Fig 10). The results indicated that the expressions of *SmDXS2*, *SmDXR*, *SmMCS*, *SmHDS*, *SmAACT*, *SmHMGS*, *SmHMGR*, *SmFPS*, *SmGGPS*, *SmSQS*, *SmCPS1*, and *SmKSL* were specifically upregulated in N0 and Nl compared to that in Nf at 45 DAT. However, the expressions of *SmAACT*, *SmHMGR*, *SmFPS*, and *SmSQS* were not significantly different among the N0, Nl and Nf treatments at 60 DAT. In addition, the expressions of the genes involved in the MEP and MVA pathways, including *SmDXS2*, *SmDXR*, *SmMCS*, *SmHDS*, and *SmHMGS*, were significantly higher in N0 than in Nl and Nf. Moreover, the expressions of downstream genes, such as *SmGGPS*, *SmCPS1*, and *SmKSL*, were lower under Nl treatment than under N0 and Nf. Further, the expressions of all four genes in the MEP pathway (*SmDXS2*, *SmDXR*, *SmMCS*, and *SmHDS*) and the downstream genes (*SmGGPS*, *SmCPS1*, and *SmKSL*) were higher in N0 and Nl than in Nf at 75 DAT. Moreover, the expression levels of all three MVA pathway genes (*SmAACT*, *SmHMGS*, and *SmHMGR*) and *SmFPS* were similar with no significant difference among N0, Nl, and Nf (Fig 10). The transcription levels of these genes, as analysed by q-PCR, were similar to those in the RNA-seq data (S5 Fig). A total of 12 genes were selected for designing gene-specific primers for qPCR analysis (S6 Fig). A linear regression suggested a good correlation between the qPCR results and the RNA-seq data from transcription with an overall correlation coefficient (R = 0.649); therefore, RNA-seq data of transcription in the roots under N0, Nl and Nf treatments were shown to be accurate and reliable by qPCR. Consequently, this study showed that nitrogen-free and low-nitrogen stress upregulated the expressions of the *SmDXR*, *SmDXS2*, *SmMCS*, *SmHDS*, *SmAACT*, *SmHMGS*, *SmHMGR*, *SmFPS*, *SmGGPS*, *SmSQS*, *SmCPS1*, *SmKSL* genes.

### Transcription factor analysis

Transcription factors may play important role in the middle and later stages of the growth of *S*. *miltiorrhiza* plant under N-free stress.Transcription factor analysis revealed that 81 classes of transcription factors showed dynamic changes of *S*. *miltiorrhiza* roots in N-free stress compared with those from normal N. The Top 15 transcription factors of the TF family were C_3_H, MADS, FAR1, bHLH, TRAF, MYB-related, NAC, PHD, SET, Orphans, FHA, AP2-EREBP, SNF2, HB and MYB. 32 types of transcription factor were down-regulated in *S*. *miltiorrhiza* roots from N-free stress, of which the expression levels of genes in the AP2/ERF, MYB, MYB-releted and WRKY families were significantly up-regulated at 60 and 75 DAT. Whereas, 26 types of transcription factor were down-regulated in *S*. *miltiorrhiza* roots from N-free stress, which of genes in the C_3_H FAR1and MADS classes showed significantly down-regulation (S2 File).

### Metabolic analysis *S*. *miltiorrhiza* in response to N starvation stress

To examine the networks between transcriptomes and metabolomes, we investigated the metabolites of *S*. *miltiorrhiza* under Nl and Nf at 45, 60, and 75 DAT using GC/TOF-MS. Orthogonal projection to latent structures-discriminate analysis (OPLS-DA) and principal component analysis (PCA) were applied to investigate differences in metabolites between the Nl and Nf at different periods. The results showed a significant difference between the Nl and Nf treatments, and the OPLS-DA score plots for M7-9 showed that Nl and Nf samples from the three periods were significantly divided into two groups according to PLS components 1 and 2. Evident differences were observed between Nf and Nl treatments at 45 DAT (R2Y = 0.995, Q2 = 0.772), 60 DAT (R2Y = 0.993, Q2 = 0.853), and 75 DAT (R2Y = 0.994, Q2 = 0.765) (S7 Fig, S11 Table). The results indicated that the metabolite profile of *S*. *miltiorrhiza* roots changed when exposed to low-N stress. Greater VIP value indicated greater contribution to the difference. The compounds with a VIP value greater than 1 are listed in S10 Table. Changes in gene expression or metabolite accumulation were indicated as fold change between the Nl and Nf treatments. Between the Nl and Nf, there were significant differences in metabolites including L-glutamic acid, 4-hydroxyphenylacetic acid, N-Acetyl-L-leucine 1, 4-hydroxymandelonitrile 2, methyl phosphate, 1,3-cyclohexanedione 2, pyrophosphate 3, α-aminoadipic acid. Most of the upregulated metabolites belong to the metabolic or biosynthesis pathways of secondary metabolites, ABC transporters, arginine, proline, butanoate, amino acids, C5-branched dibasic acid, glyoxylate and dicarboxylate metabolism, 2-oxocarboxylic acid, alanine, aspartate, glutamate, carbon, and arginine (S12 Table).

## Discussion

Nitrogen plays an essential role in the growth and developmental processes of traditional Chinese medicine herbs, and appropriate N applications can promote the growth of herbs, increase their yields, and improve the quality of the resulting medicine [40]. N is beneficial to the synthesis and accumulation of alkaloids. Reasonably increasing N during the cultivation of herbs, such as *Leonurus heterophyllus* and *Pinellia ternata* could ensure the quality of herbal medicine [73,74]. At present, artificially cultivated *S*. *miltiorrhiza* has become the main source of the medicine Danshen in the market of China. Many studies have indicated that moderate amounts of N can increase root diameter and root yield and can promote the accumulation of its medicinally active components, such as tanshinones [53]. In this study, high or normal-N concentrations increased root biomass and root diameter in *S*. *miltiorrhiza* (Fig 2), it can be seen that the roots of *S*. *miltiorrhiza* under Nf conditions were bigger and had larger diameters, and that the aboveground plant organs were also relatively bigger, however, the root epidermis area was smaller. The fresh and dry weights and the aboveground parts of the plants under the Nf treatment were observably higher than those of the plants under the N0 treatment, but there was no remarkable difference in the fresh and dry weights between the Nf and Nl conditions.

These results indicated that nitrogen greatly promoted root biomass. Moreover, compared to normal N conditions, low N had no obvious effect on root growth, whereas N-free conditions seriously inhibited root growth and development, which was consistent with the results of previous studies [53]. It is well known that tanshinones are mainly distributed in the root epidermis; therefore, root size in *S*. *miltiorrhiza* is very important because it is related to the content and quality of tanshinones. Many reports have shown that the diameter of the root is positively related to total tanshinones, and that bigger roots had fewer tanshinones [75,76]. Moreover, the characteristic pigmentation is imparted by the tanshinones [7]. In this study, it was apparent that the root colour in the no and low nitrogen treatments (N0 and Nl) was deeper than that of the normal-nitrogen treatment (Nf). This study also confirmed that root tanshinone contents, including those of TS I, CTS, TS IIA, and TTS, were significantly lower in the Nf treatment than in the N starvation (N0 and Nl) treatments (Fig 3). The results of this study were in line with the research reports of He et al. (2010) and Yang et al. (2010) [75,76]. Collectively, these results confirm that low nitrogen conditions promote the formation and accumulation of tanshinones to a certain extent.

According to previous studies, tanshinones are a kind of non-olefinic diterpenoid compounds, which are an important source of bioactive components in roots of *S*. *miltiorrhiza*, and have good therapeutic effects and pharmacological activity [2]. IPP and DMAPP, known as two common precursors of plant terpenoids, are synthesised through the MVA and MEP pathways; *SmAACT*, *SmHMGS*, *SmHMGR* are genes for important enzymes in the MVA pathway, whereas *SmDXS2*, *SmDXR*, *SmMCS* and *SmHDS* are genes for important enzymes in the MEP pathway, and *SmGGPS*, *SmCPS1* and *SmKSL* are the major (MVA) downstream genes [12]. Previously some studies have identified most of the genes related to the biosynthesis of tanshinones and phenolic acids [8,77–80], and these were also found in our RNA-seq transcriptome dataset (S4 and S5 Tables, S8 Fig).

In this study, the expressions of the genes that encode the key enzymes in the MVA and MEP pathways, including those of *SmDXS2*, *SmDXR*, *SmMCS*, *SmHDS*, *SmAACT*, *SmHMGS*, *SmHMGR*, *SmFPS*, *SmGGPS*, *SmSQS*, *SmCPS1*, and *SmKSL*, were upregulated under N0 and Nl conditions at the early development period (45 DAT). At 60 DAT under N0, Nl and Nf conditions, the expressions of *SmAACT*, *SmHMGR*, *SmFPS*, and *SmSQS* showed no obvious differences; however, the expressions of genes in the MEP and MVA pathways, including those of *SmDXS2*, *SmDXR*, *SmMCS*, *SmHDS*, and *SmHMGS*, were significantly higher in N0 than in Nl and Nf, and the expressions of downstream genes, such as *SmGGPS*, *SmCPS1*, and *SmKSL*, were slightly lower under Nl than under N0 and Nf conditions. However, at a later development period (75 DAT), the expressions of all four genes in the MEP pathway (*SmDXS2*, *SmDXR*, *SmMCS*, and *SmHDS*) and the (MVA) downstream genes (*SmGGPS*, *SmCPS1*, and *SmKSL*) were higher in N0 and Nl than in Nf (Figs 6 and S8). Consequently, these results suggested that N stress induced tanshinone synthesis and that the expressions of the genes involved in tanshinone synthesis were higher under N-free conditions than in the presence of N during the entire growth and development period. However, low N levels promoted tanshinone synthesis in the early and late stages of plant growth. These results also indicated that N starvation and low-N stress stimulated the genes involved in tanshinone biosynthesis, and then promoted tanshinone accumulation in the roots of *S*. *miltiorrhiza*, which was consistent with the finding of increased metabolite contents (S9 Fig, S12 Table).

In the early stage of growth, tanshinones (TS I, TS IIA, and CTS) began to form and accumulate [81], but in the middle stage, tanshinone production tended to be limited because of the growth of *S*. *miltiorrhiza*. In the later stage, a large of amount tanshinones formed and accumulated [82]; therefore, it was implied that the demand for N differed at different stages of growth. The effect of N on the synthesis of tanshinones and other metabolites was inconsistent. Therefore, it can be inferred that early exposure to N-free or low-N conditions promoted the formation and accumulation of metabolites through the MVA and MEP pathways. N supply increased the biomass of *S*. *miltiorrhiza* roots and inhibited the formation and accumulation of metabolites such as tanshinones. In the mid-term, because of other biological processes, tanshinones and other metabolites were in a biologically stable period [13,75]. At the later stage of quantitative development, the genes of tanshinones and other metabolites were active again, leading to metabolite generation and accumulation. At this time, compared with the sufficient nitrogen environment, low nitrogen environment promotes the formation and accumulation of tanshinone and other metabolites. Furthermore, in the study, under nitrogen starvation conditions, glutamine synthetase activity increases (Fig 8), which could be beneficial to increase nitrogen utilization. Nitrogen metabolism-related genes are significantly up-regulated (Fig 9), which could be conducive to improving the efficiency of nitrogen transfer and assimilation [27,53].Transcription factors may play important role in the middle and later stages of the growth of *S*. *miltiorrhiza* plant under N-free stress (S2 File).

In this study, analyses of gene enrichment of the GO and KEGG metabolic pathways based on the transcriptomic and metabolomic data showed that the low-nitrogen condition greatly affected the expressions of genes in the metabolic pathways for tanshinone and other substances,the results suggested they may play important role in the middle and later stages of the growth of *S*. *miltiorrhiza* plant under N-free stress. For example, topGO enrichment results showed that the biological processes of “phosphate ion transmembrane transport”, “phosphate ion transport”, “response to chitin”, and “response to wounding” were obviously enriched by downregulated DEGs in N0 vs. Nf and N0 vs. Nf at 45, 60, and 75 DAT. The results suggested that N starvation (N0 and Nl) decreased the absorption and transport of phosphate during almost the whole development period. Moreover, the processes “response to water deprivation” and “wounding and deprivation progresses” were significantly enriched by downregulated DEGs in Nl vs. Nf at 75 DAT, indicating that low-N conditions affected the abilities of *S*. *miltiorrhiza* to tolerate other kinds of environmental stress.

The study also showed that the nitrate reductase activity in the roots under nitrogen starvation is reduced, while the glutamine synthetase activity is increased, compared with the normal nitrogen supply (Fig 8). Compared with the normal nitrogen supply, under nitrogen starvation treatment, the transcription (FPKM) expressions of GS family genes (GS1, GS2), Nar1 and nitrate transporter genes NRT2 family (NRT2.1, NRT2.5, NRT2.6, NRT2.7) in roots, was significantly up-regulated (P<0.01) (Fig 9). Furthermore, “glucose-6-phosphate transport” and “phosphoenolpyruvate transport” were enriched by upregulated DEGs under N0 treatment at 45 DAT, strongly suggesting that N-free condition promoted glycolysis and gluconeogenesis at the early stage of growth. The term “transcription, DNA-templated” was markedly enriched by upregulated DEGs under N0 treatment at 60 and 75 DAT, which suggested that showed that under low-N conditions, lipid and amino acid metabolisms and C metabolism were strongly affected. In addition, cholesterol metabolism, lipid catabolism, and nucleoside or amino acid transport were enriched significantly by upregulated DEGs under the Nl condition at 45, 60, and 75 DAT (S3 Fig).

Among cellular components, “integral component of plasma membrane”, “plant−type vacuole membrane”, “integral component of membrane”, and “plasma membrane” were enriched by upregulated DEGs in N0 vs. Nf and Nl vs. Nf at 45, 60, and 75 DAT; nevertheless, “chloroplast” and “central vacuole” were enriched by downregulated DEGs in N0 vs Nf. These results indicated that N starvation (N0 and Nl) seriously improved plasma and vacuole membrane development, as well as inhibited chloroplast thylakoid membrane or chloroplast development (S3 Fig).

Moreover, two key enzymes, DXR and HDS, were enriched by upregulated DEGs under N starvation, suggesting their important role in tanshinone biosynthesis (S3 Fig, S7 Table). In the molecular function category, the term “phosphate ion transmembrane transporter activity” was enriched by downregulated DEGs in N0 vs. Nf and Nl vs. Nf at almost all treatments and stages except for Nl treatment at 75 DAT. Moreover, “transcription factor activity” and “sequence-specific DNA binding” were obviously enriched by upregulated DEGs in N0 vs. Nf at 60 and 75 DAT. At the same time, “mannose binding” and “mRNA methyl transferase activity” were also enriched by many upregulated DEGs in Nl vs. Nf at 60 DAT. 1-deoxy-D-xylulose-5-phosphate reductoisomerase (DXR) was significantly enriched by upregulated DEGs under N0 and Nl conditions at 45 or 60 DAT. Moreover, 4-hydroxy-3-methybut-2-en-1-yl diphosphate synthase (HDS) was upregulated under N0 treatment at 75 DAT, the results suggested the DXR and HDS key enzymes in tanshinone biosynthesis was clearly increased by N stress.

The results of the study also strongly indicated that N-free condition improved tanshinone synthesis. In this study, KEGG enrichment analysis showed that all DEGs involved in photosynthesis, nitrogen metabolism, galactose metabolism, glycerolipid metabolism, and phenylpropanoid biosynthesis were enriched after N starvation (N0 and/or Nl) at 45, 60, and 75 DAT. Under N0 and Nl conditions, terpenoid backbone biosynthesis was enriched by upregulated DEGs at all three stages.

Furthermore, These study indicated that N-free and low-N treatments had different effects on the growth and development of *S*.*miltiorrhiza* at different stages. at 60 DAT, plant hormone signal transduction and phenylpropanoid biosynthesis pathways were mostly enriched by upregulated DEGs under the N0 treatment. However, nitrogen metabolism and photosynthesis pathways were mostly enriched by downregulated DEGs under N0 or Nl conditions at the early period (45 DAT), and flavone and flavonol biosynthesis pathways were enriched by downregulated DEGs under N0 condition at 75 DAT. Simultaneously, downregulated DEGs enriched starch and sucrose metabolism pathways (Fig 7, S9 Table). Through the KEGG map, we found that N starvation mainly affected the MEP pathway at 45 and 75 DAT. Few DEGs were mapped under the Nl condition compared to that under Nf at 60 DAT, but three DEGs encoding enzymes of the MVA pathway were found to be downregulated under Nl condition. These results seemed to suggest that low-N conditions promoted tanshinone accumulation in *S*.*miltiorrhiza*. Furthermore, the genes involved in terpenoid backbone biosynthesis were upregulated (S4 Fig, S10 Table) by N starvation (N0 and Nl), which indicated that tanshinones were actively biosynthesised. DEG annotation provided valuable resources for the recognition of specific genes and signal transduction pathways that respond to low nitrogen stress, especially for investigating the biosynthesis mechanism of secondary metabolites in *S*.*miltiorrhiza*. Previous studies have shown that starch and carbohydrate degradation promotes the formation and accumulation of metabolites [83]. The results also showed that the expressions of genes involved in starch and carbohydrate metabolism were downregulated, whereas those of the genes involved in tanshinone metabolic pathways were upregulated. It can be inferred that the degradation and decline of starch and carbohydrates in *S*. *miltiorrhiza* under low-nitrogen conditions promoted the formation and accumulation of tanshinones and other substances.

Metabolic data are important for elucidating the correlation between gene functions and phenotypes [84]. We used PLS-DA and PCA to reveal metabolomic in a significant difference in the roots of *S*. *miltiorrhiza* exposed to low-N and normal-N conditions (S7 Fig), and metabolomic analysis showed Nitrogen metabolites (L-glutamic acid, 4-hydroxyphenylacetic acid, N-Acetyl-L-leucine 1, 4-Hydroxymandelonitrile 2, Methyl Phosphate, 1,3-Cyclohexanedione 2, pyrophosphate 3, α-Aminoadipic acid), and most of secondary metabolites were up-regulated (S12 Table). Moreover, through the combined analysis of transcriptome and metabolome, there is a significant correlation between the expression of tanshinone metabolic synthesis pathway and carbohydrate metabolism pathway, tanshinone metabolic synthesis pathway is up-regulated, and carbohydrate metabolism pathway substances are down-regulated (S10 Table, S10 Fig). The results showed that most of the upregulated metabolite compounds were involved in the metabolism and biosynthesis of secondary metabolites. Furthermore, Gene and metabolite network in cytoscape showed metabolites with significantly differences among the samples were extracted. Correlation coefficient values were calculated between normalised read counts of the genes and concentrations of the metabolites. Then gene-metabolite with correlation coefficient values above 0.9 or below -0.9 were used to construct the gene-metabolite network (S3 File). Consequently, it can be reasoned that low-nitrogen stress could stimulate the accumulation of secondary metabolites by *S*. *miltiorrhiza*.

## Conclusion

This study showed a whole-transcriptome profile of *S*. *miltiorrhiza* root in response to low-N stress. The present study unveiled that low nitrogen stress affected and improved tanshinone content in the roots of *S*. *miltiorrhiza* by regulating the expression of genes involved in the biosynthesis pathways of secondary metabolites, such as tanshinones, diterpenoids, and terpenoid backbone. Therefore, low-N treatment can be considered an effective approach for increasing the production of tanshinones in *S*. *miltiorrhiza* root culture systems. However, low-N stress would not promote the root biomass. Therefore, the appropriate nitrogen nutrition should be applied in the cultivation of *S*. *miltiorrhiza* to ensure the yield of *S*. *miltiorrhiza* and improve the production of tanshinones.

## Supporting information

S1 Fig Number and distribution of up- and downregulated genes after treatment with different N levels in N0, Nl, and Nf. (a) 45 DAT vs. 60 DAT, (b) 45 DAT vs. 75DAT, (c) 60 DAT vs. 75 DAT; DAT: Days after transplanting.
(TIF)

S2 Fig Histogram of GO classification of DEGs in N0 vs. Nf, Nl vs. Nf, and N0 vs. Nl at 45 and 60 days after transplanting (DAT). Blue represents all the unigenes annotated in each subcategory, red represents all DEGs annotated in each subcategory. The right y-axis represents the number of genes annotated in each subcategory. The left y-axis represents the percentage of annotated unigenes or DEGs in that main category. (a, b) N0 vs. Nf, (c, d) Nl vs. Nf, (e, f) N0 vs. Nl.
(TIF)

S3 Fig Enrichment analysis of the GO pathways in N0 vs. Nf, Nl vs. Nf, and N0 vs. Nl at 75 days after transplanting (DAT). All DEGs, including up- and downregulated DEGs, were distributed in the GO pathways. The rich factor was the ratio of the number of DEGs in a pathway to the number of all genes involved in this pathway. The degree of gene enrichment was enhanced with increasing rich factor and decreasing (Q value). The Q value is the rectified P-value (FDR). (a) All DEGs in N0 vs. Nf, (b) all DEGs in Nl vs. Nf, (c) all DEGS in N0 vs. Nl, (d) upregulated DEGs in N0 vs. Nf, (e) upregulated DEGs in Nl vs. Nf, (f) upregulated DEGs in N0 vs. Nl, (g) downregulated DEGs in N0 vs. Nf, (h) downregulated DEGs in Nl vs. Nf, (i) downregulated DEGs in N0 vs. Nl.
(TIF)

S4 Fig KEGG enrichment analysis of terpenoid backbone biosynthesis pathway diagram. (a) N0 vs. Nf at75 days after transplanting (DAT); (b) Nl vs. Nf at 75 days after transplanting (DAT).
(TIF)

S5 Fig Heat map describing the expression profile of tanshinone biosynthesis-related genes from the MEP and MVA pathways in the root of S. miltiorrhiza treated with low and normal nitrogen levels (a: N0, Nl, and Nf at 45, 60, and 75 days after transplanting (DAT)). qRT-PCR validation of differential expression (b: N0 at 45, 60, and 75 DAT; c: Nl at 45, 60, and 75 DAT; Nf at 45, 60, and 75 DAT).
(TIF)

S6 Fig qRT-PCR verification of differential gene expression. (a) A total of 12 genes; the data for RNA-seq in R; the data for qRT-PCR in q. Bars indicate ± SE (n = 3). (b) Comparison of the gene expression ratios obtained from RNA-seq data and qRT-PCR. RNA-seq log2 value of the expression ratios.
(TIF)

S7 Fig Score (a, c) and loading (b, d) plot of the PLS-DA model based on the metabolic profile of S. miltiorrhiza root-based metabolic compounds in Nl vs. Nf at (a, b) 45 and (c, d) 60 days after transplanting (DAT).
(TIF)

S8 Fig FPKM from the data for RNA-seq of genes in Biosynthesis of tanshinones. (TIF)

S9 Fig KEGG enrichment analysis of metabolic pathways from the metabolomes of *S*. *miltiorrhiza* in Nl (75 days after transplanting (DAT)) vs. Nf (75 DAT). Red/blue dots represent the differentially expressed compounds. Bright red represents upregulation, bright blue represents downregulation.
(TIF)

S10 Fig The analysis of metabolites expression through the combination of transcriptome and metabolome. Red represents up-regulation and blue represents down-regulation.
(TIF)

S1 Table Summary of the RNA-seq data collected from N0, Nl, and Nf at each of the three selected *S*. *miltiorrhiza* developmental stages. N0: No nitrogen; Nl: Low nitrogen level; Nf: Normal nitrogen level; DAT: Days after transplanting.
(XLSX)

S2 Table Unigene length data for *S*. *miltiorrhiza* mapped to the reference transcriptome. (DOCX)

S3 Table Differentially expressed (common) genes in N0 vs. Nl, N0 vs. Nf, and Nl vs. Nf at 45, 60, and 75 days after transplanting (DAT). (DOCX)

S4 Table Differentially expressed (common) genes in N0 vs. Nf and Nl vs. Nf at 45, 60, and 75 days after transplanting (DAT). (XLSX)

S5 Table Data for GO enrichment DEG statistics from *S*. *miltiorrhiza* mapped to the reference transcriptome in N0 vs. Nf, Nl vs. Nf, and N0 vs. Nf at 45, 60, and 75 days after transplanting (DAT). (XLSX)

S6 Table Top 20 GO enrichment pathways involved in N0 vs. Nf, Nl vs. Nf, and N0 vs. Nl at 45, 60, and 75 days after transplanting (DAT). (DOCX)

S7 Table Data for KEGG enrichment pathway statistics from *S*. *miltiorrhiza* mapped to the reference transcriptome in N0 vs. Nf, Nl vs. Nf, and N0 vs. Nl at 45, 60, and 75 days after transplanting (DAT). (XLSX)

S8 Table Top 20 KEGG enrichment pathways involved in N0 vs. Nf, Nl vs. Nf, and N0 vs. Nl at 45, 60, and 75 days after transplanting (DAT). (DOCX)

S9 Table Tanshinone Synthetic (KEGG) Pathway Gene Expression in N0 vs. Nf and Nl vs. Nf at 75 days after transplanting (DAT). (XLSX)

S10 Table OPLS-DA model cumulative interpretation rate. (DOCX)

S11 Table Differences in the characteristics of metabolites in the roots of *S*. *miltiorrhiza* between treatment with low and normal nitrogen levels. (DOCX)

S12 Table Primer sequences for real-time PCR of *S*. *miltiorrhiza*. (XLSX)

S1 File Length distributions of all unigenes. (PDF)

S2 File Transcription factor expression of the root of S. miltiorrhiza in N0 vs. Nf at 45 (a), 60(b) and 75 (c) days after transplanting (DAT). (d) TF family of the root of S. miltiorrhiza in the RNA-seq data.
(ZIP)

S3 File Gene and metabolite network in cytoscape. (PDF)
