# Supplementary figures and images for: Integrative transcriptomic and metabolomic analyses unveil tanshinone biosynthesis in Salvia miltiorrhiza root under N starvation stress
Source: PLoS One. 2022 Aug 25;17(8):e0273495. doi: 10.1371/journal.pone.0273495 (PMC9409544; doi:10.1371/journal.pone.0273495)

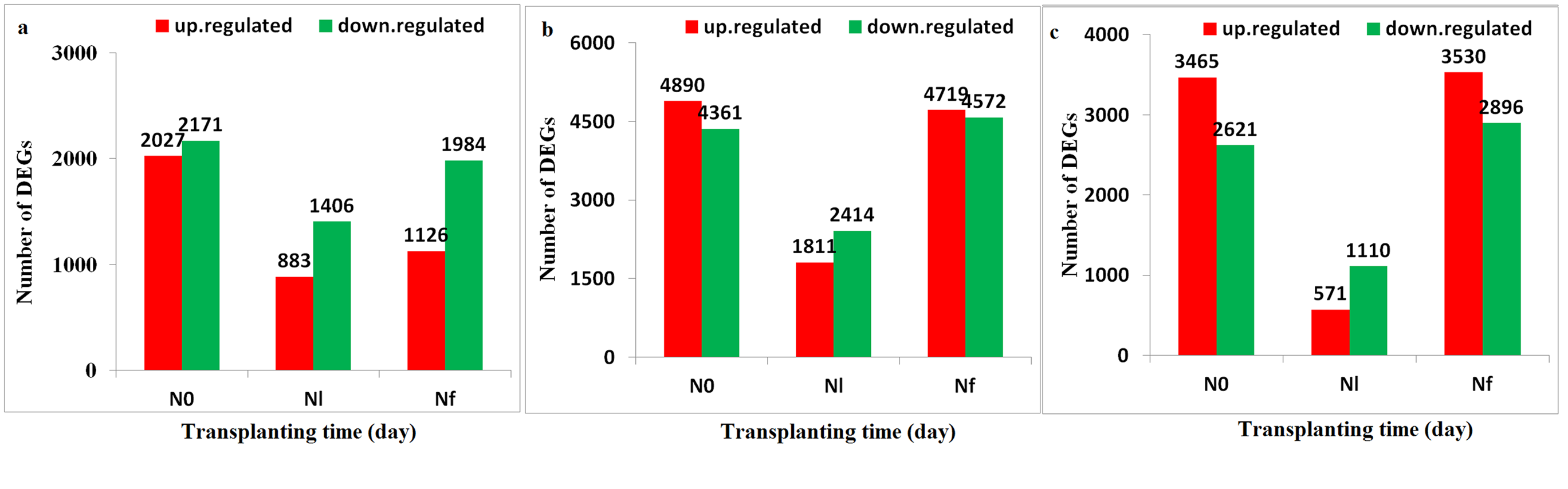

Supplement: S1 Fig — (a) 45 DAT vs. 60 DAT, (b) 45 DAT vs. 75DAT, (c) 60 DAT vs. 75 DAT; DAT: Days after transplanting. (TIF) [file pone.0273495.s001.tif]

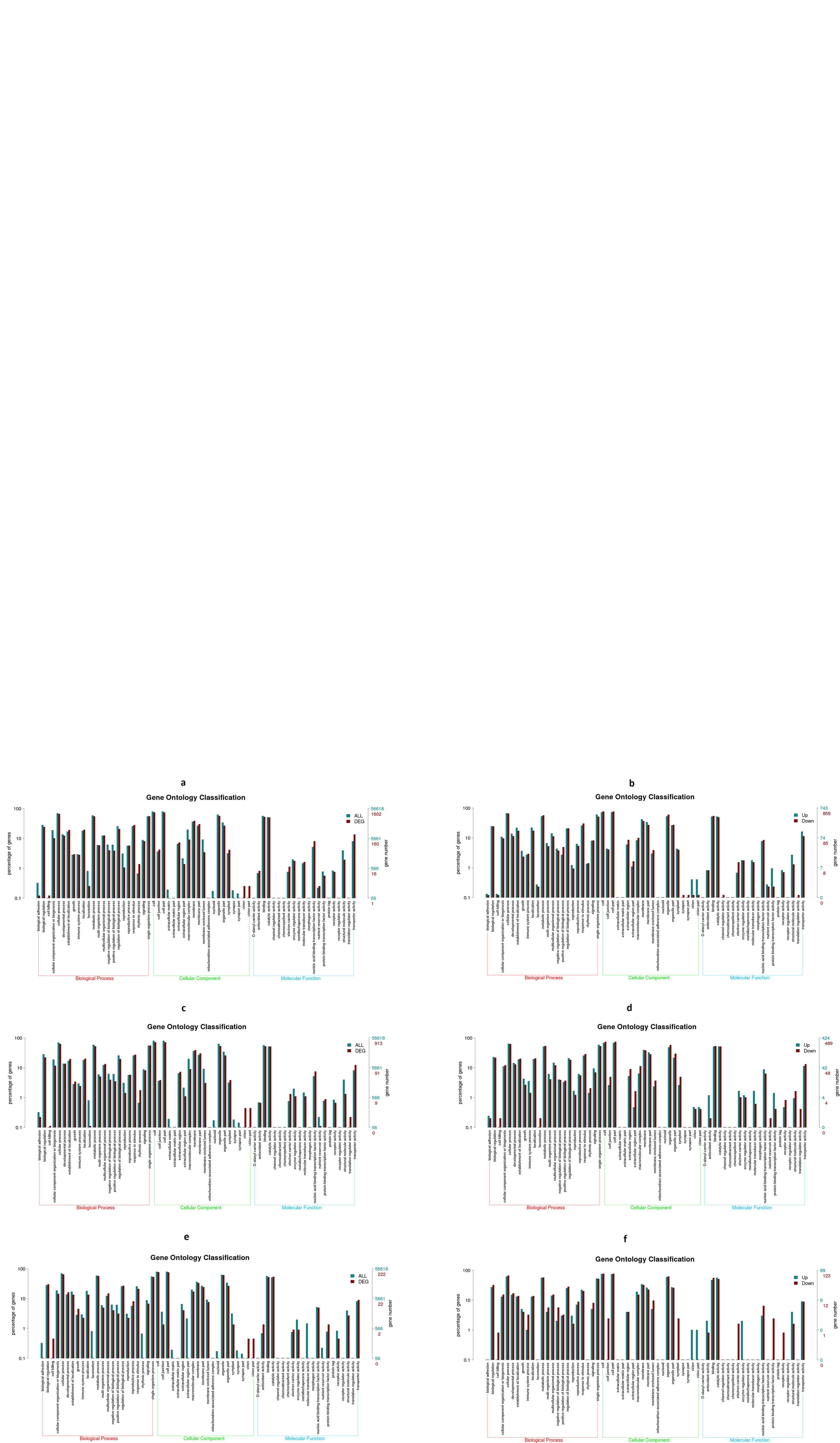

Supplement: S2 Fig — Blue represents all the unigenes annotated in each subcategory, red represents all DEGs annotated in each subcategory. The right y-axis represents the number of genes annotated in each subcategory. The left y-axis represents the percentage of annotated unigenes or DEGs in that main category. (a, b) N0 vs. Nf, (c, d) Nl vs. Nf, (e, f) N0 vs. Nl. (TIF) [file pone.0273495.s002.tif]

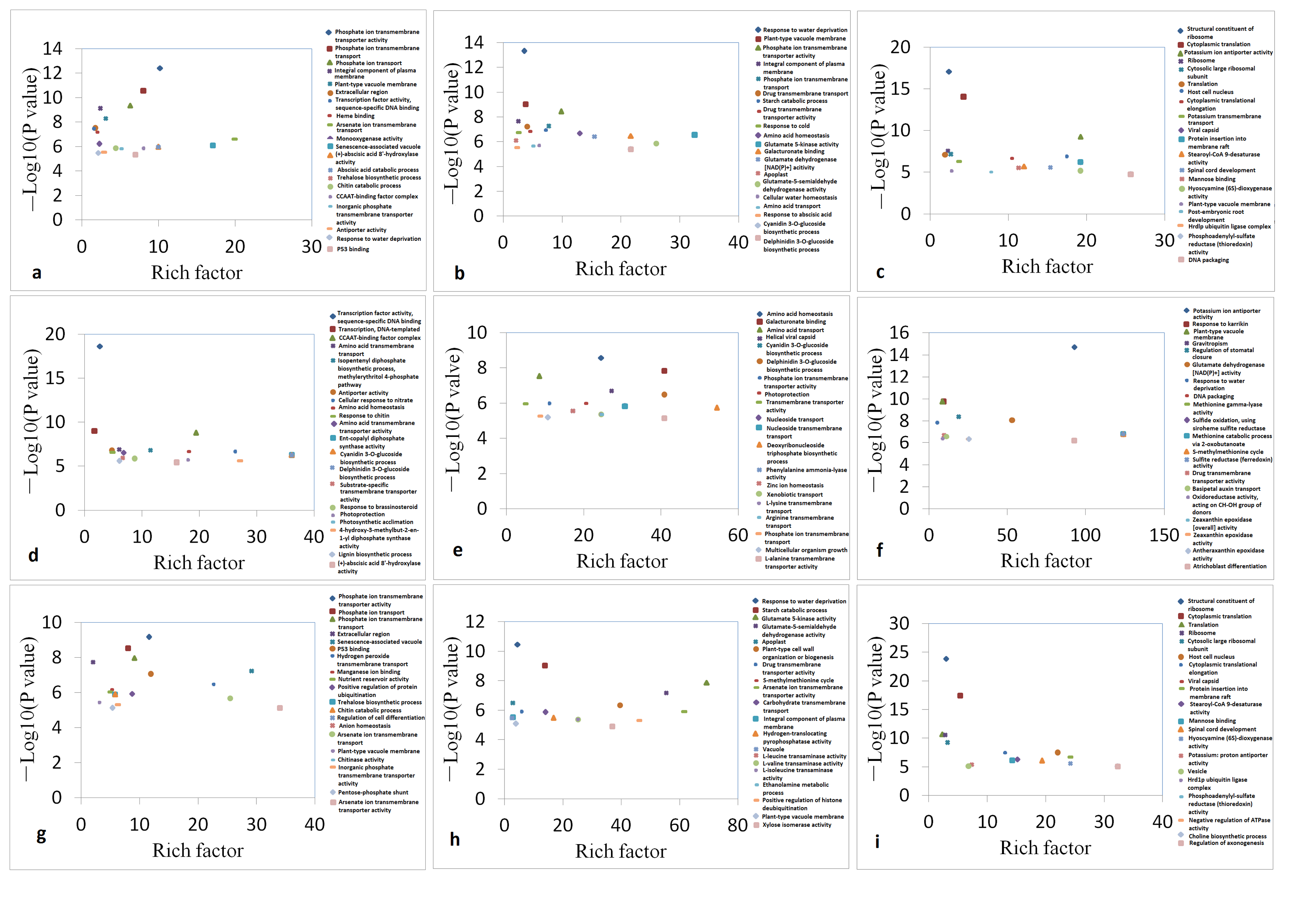

Supplement: S3 Fig — All DEGs, including up- and downregulated DEGs, were distributed in the GO pathways. The rich factor was the ratio of the number of DEGs in a pathway to the number of all genes involved in this pathway. The degree of gene enrichment was enhanced with increasing rich factor and decreasing (Q value). The Q value is the rectified P-value (FDR). (a) All DEGs in N0 vs. Nf, (b) all DEGs in Nl vs. Nf, (c) all DEGS in N0 vs. Nl, (d) upregulated DEGs in N0 vs. Nf, (e) upregulated DEGs in Nl vs. Nf, (f) upregulated DEGs in N0 vs. Nl, (g) downregulated DEGs in N0 vs. Nf, (h) downregulated DEGs in Nl vs. Nf, (i) downregulated DEGs in N0 vs. Nl. (TIF) [file pone.0273495.s003.tif]

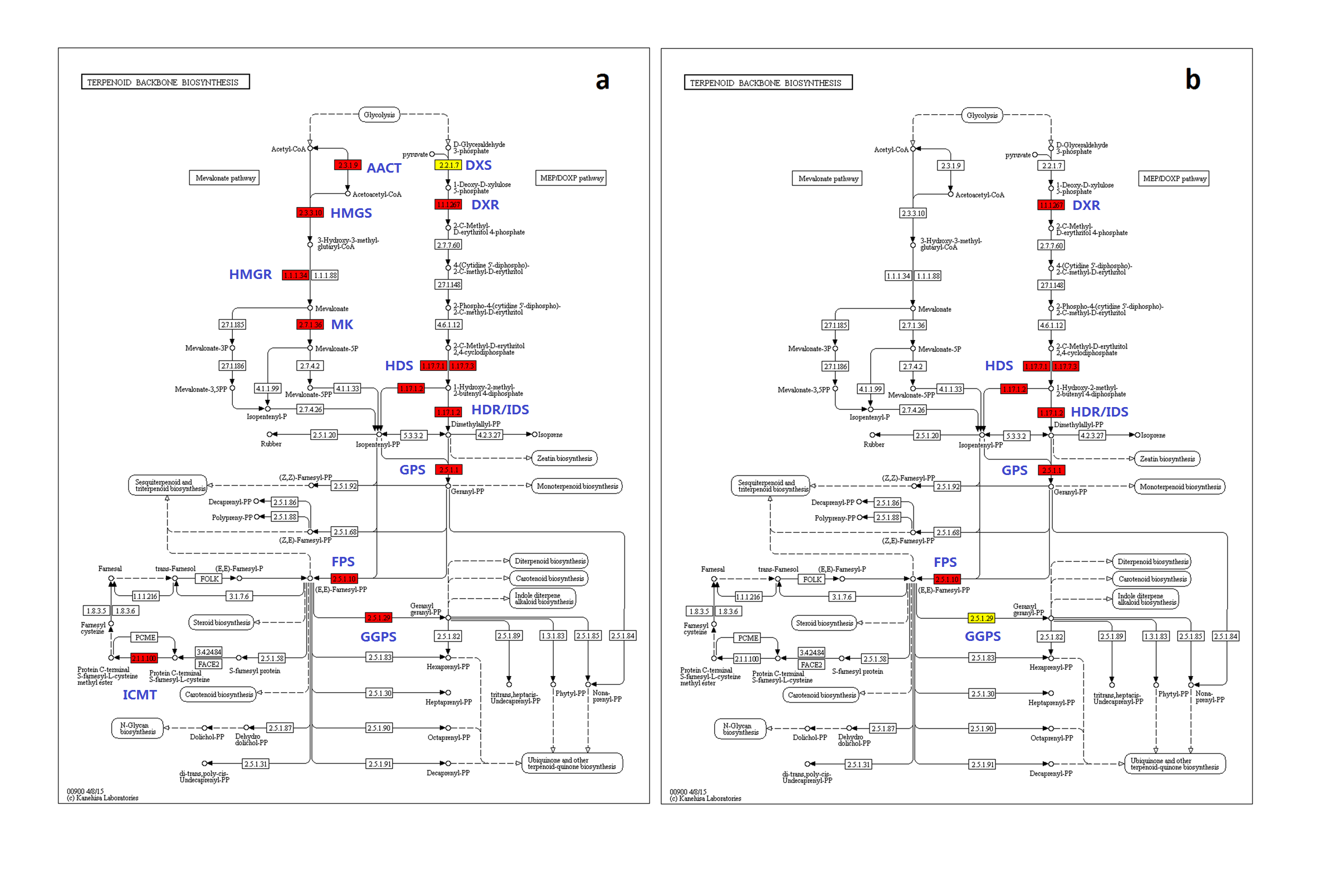

Supplement: S4 Fig — (a) N0 vs. Nf at75 days after transplanting (DAT); (b) Nl vs. Nf at 75 days after transplanting (DAT). (TIF) [file pone.0273495.s004.tif]

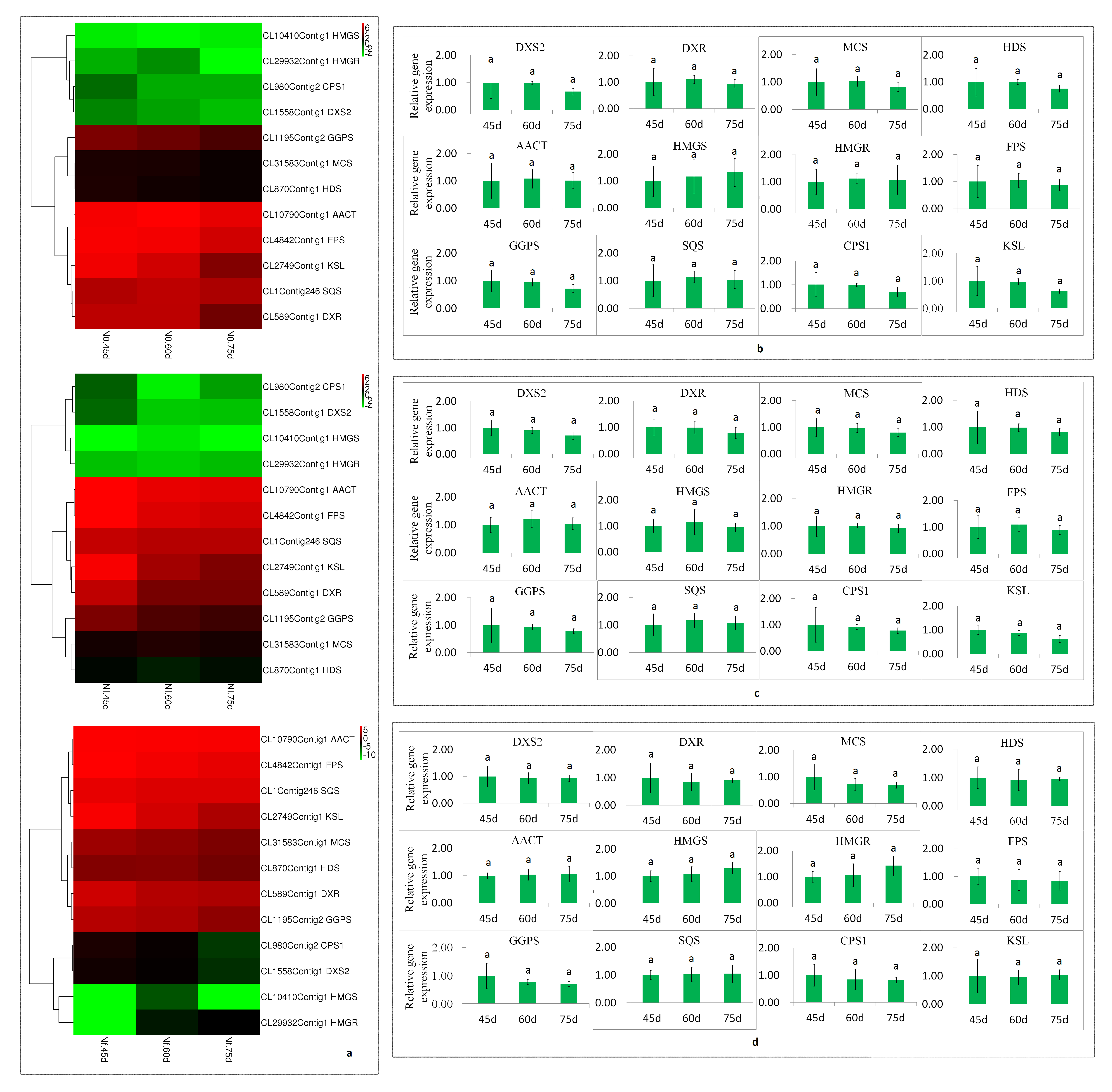

Supplement: S5 Fig — qRT-PCR validation of differential expression (b: N0 at 45, 60, and 75 DAT; c: Nl at 45, 60, and 75 DAT; Nf at 45, 60, and 75 DAT). (TIF) [file pone.0273495.s005.tif]

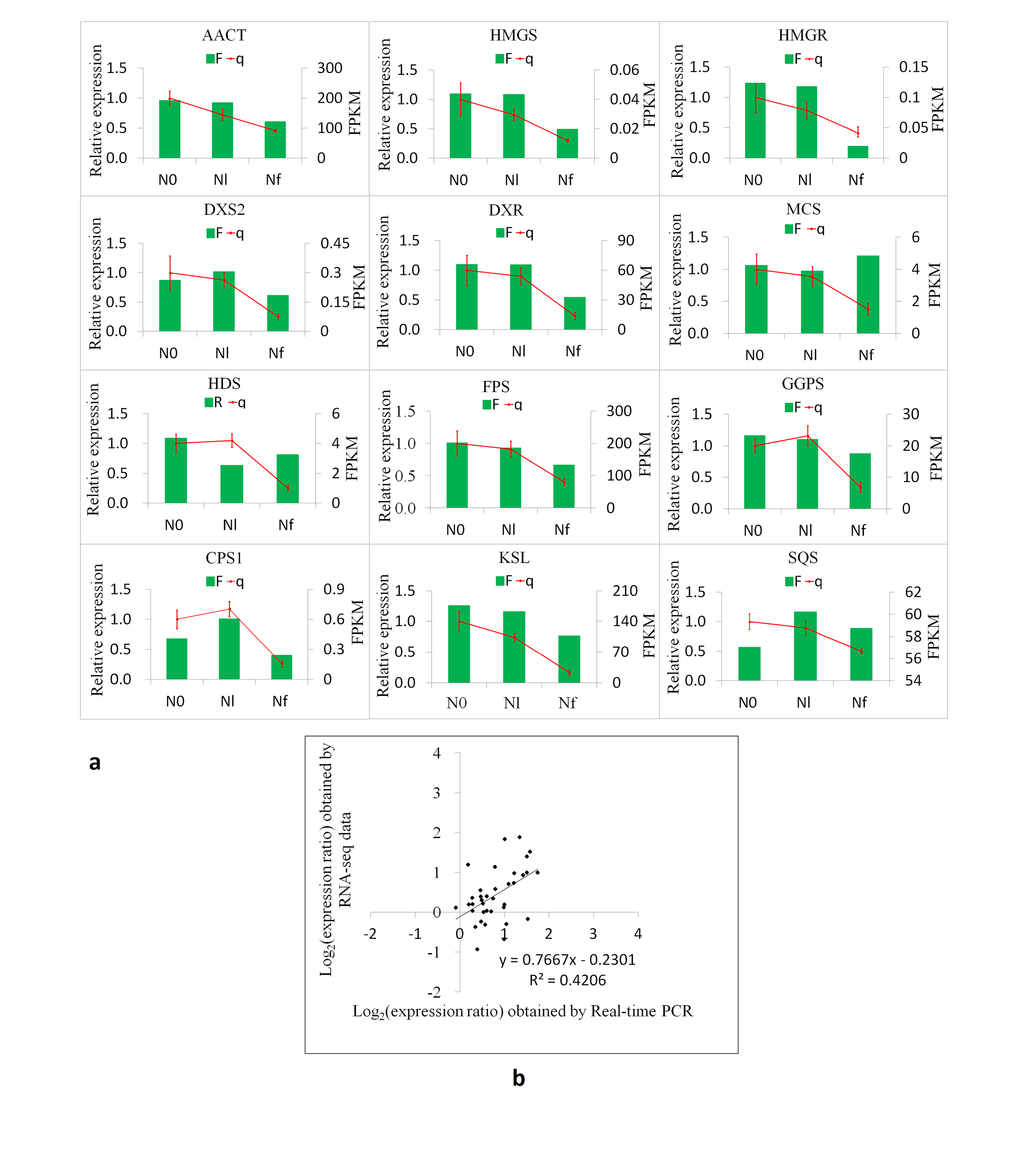

Supplement: S6 Fig — (a) A total of 12 genes; the data for RNA-seq in R; the data for qRT-PCR in q. Bars indicate ± SE (n = 3). (b) Comparison of the gene expression ratios obtained from RNA-seq data and qRT-PCR. RNA-seq log2 value of the expression ratios. (TIF) [file pone.0273495.s006.tif]

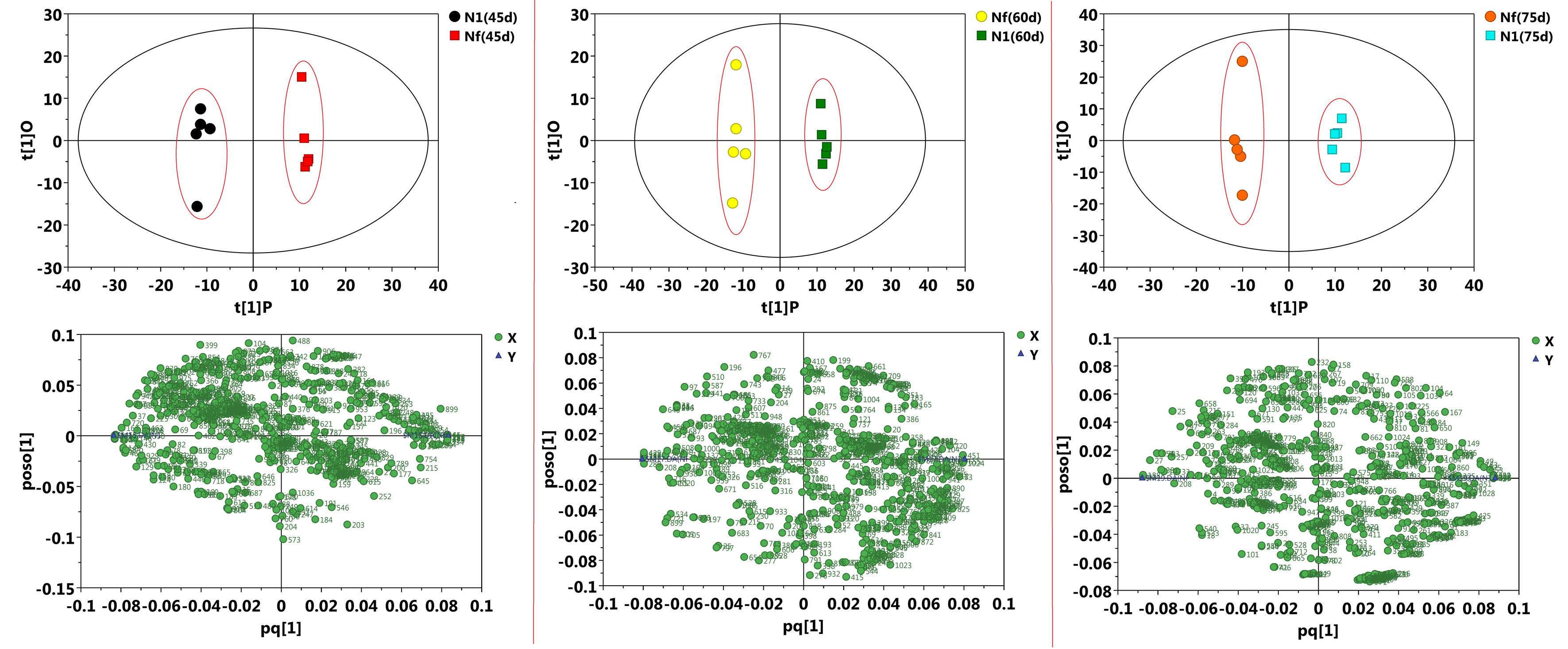

Supplement: S7 Fig — Score (a, c) and loading (b, d) plot of the PLS-DA model based on the metabolic profile of S. miltiorrhiza root-based metabolic compounds in Nl vs. Nf at (a, b) 45 and (c, d) 60 days after transplanting (DAT). (TIF) [file pone.0273495.s007.tif]

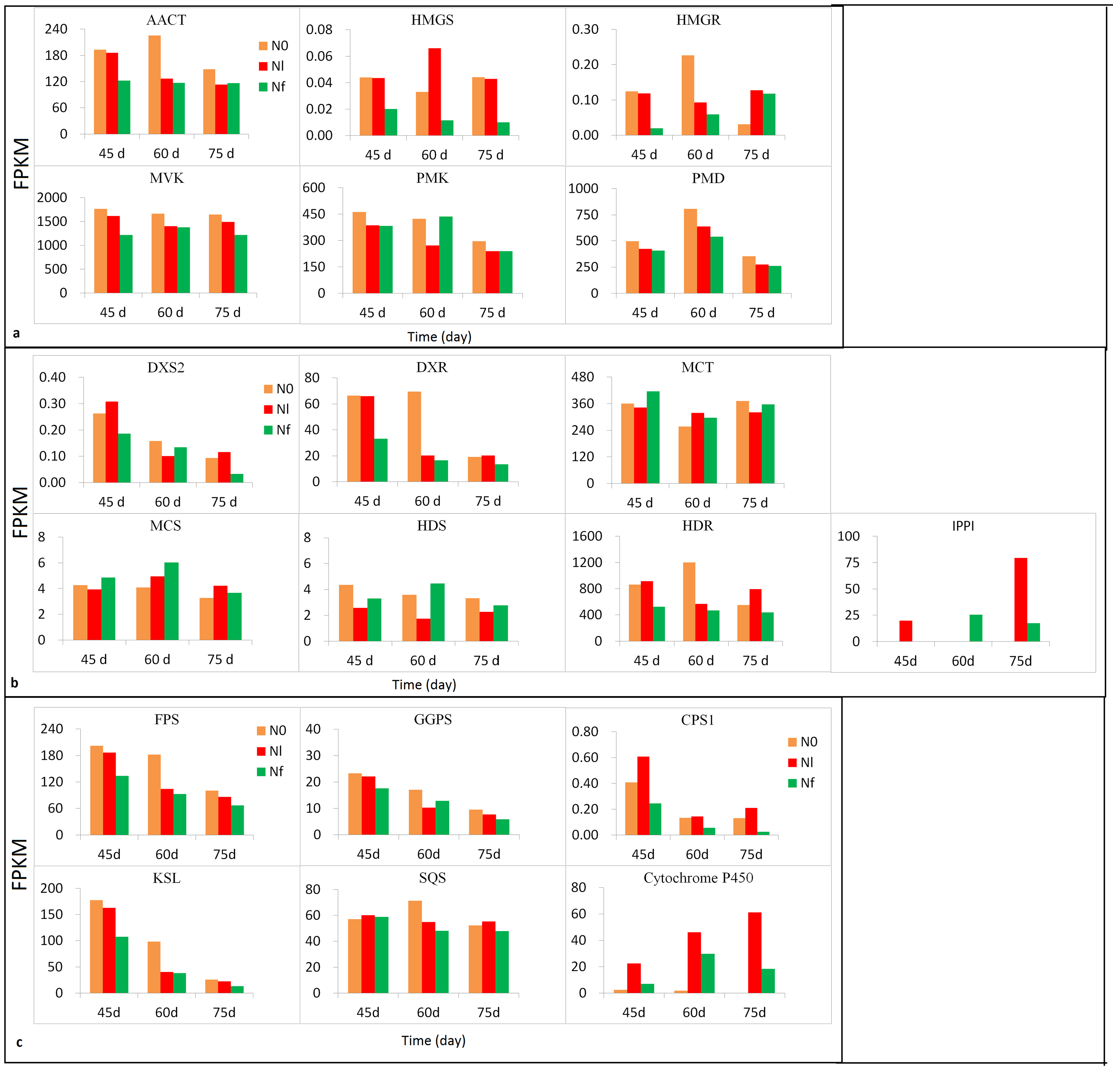

Supplement: S8 Fig — (TIF) [file pone.0273495.s008.tif]

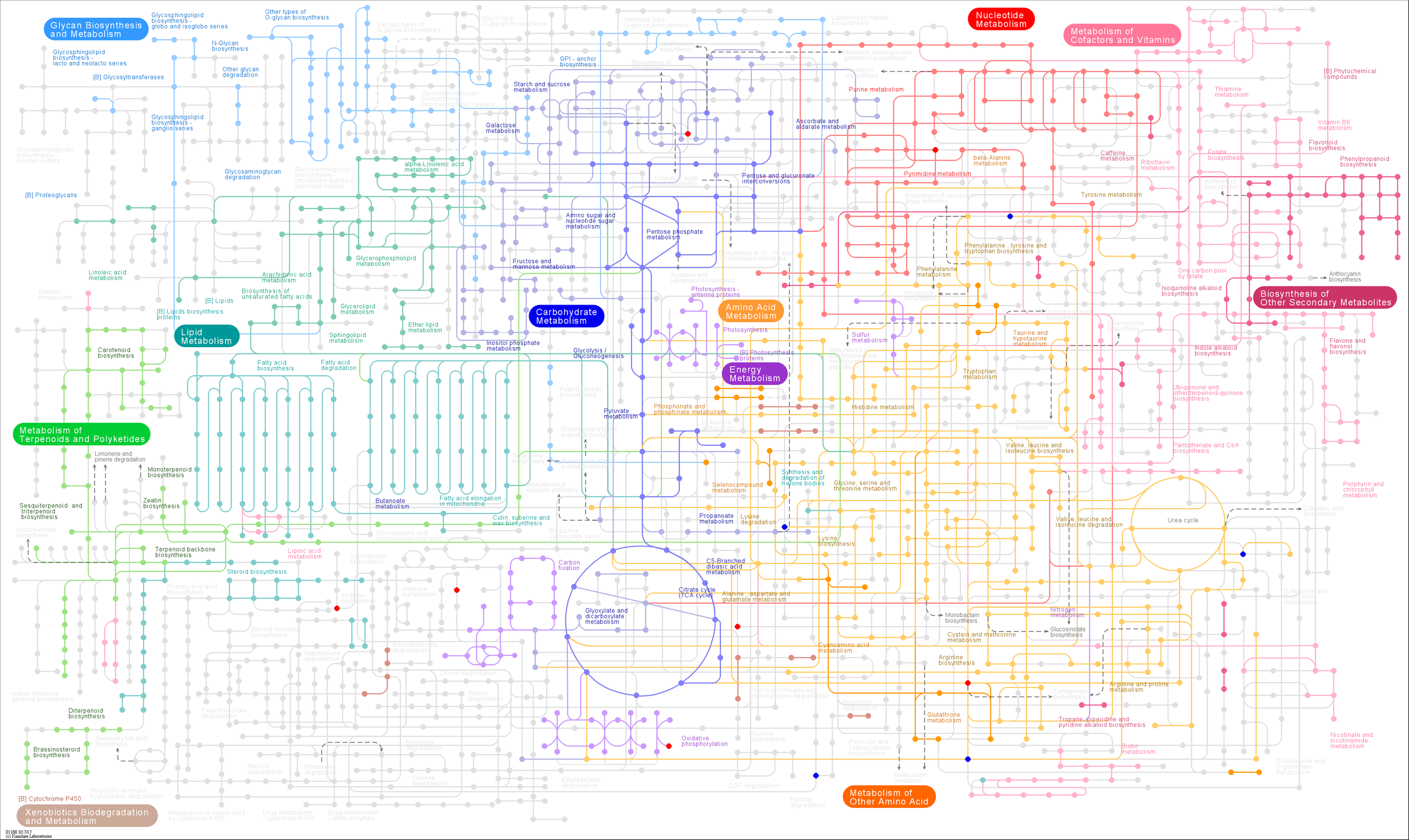

Supplement: S9 Fig — Red/blue dots represent the differentially expressed compounds. Bright red represents upregulation, bright blue represents downregulation. (TIF) [file pone.0273495.s009.tif]

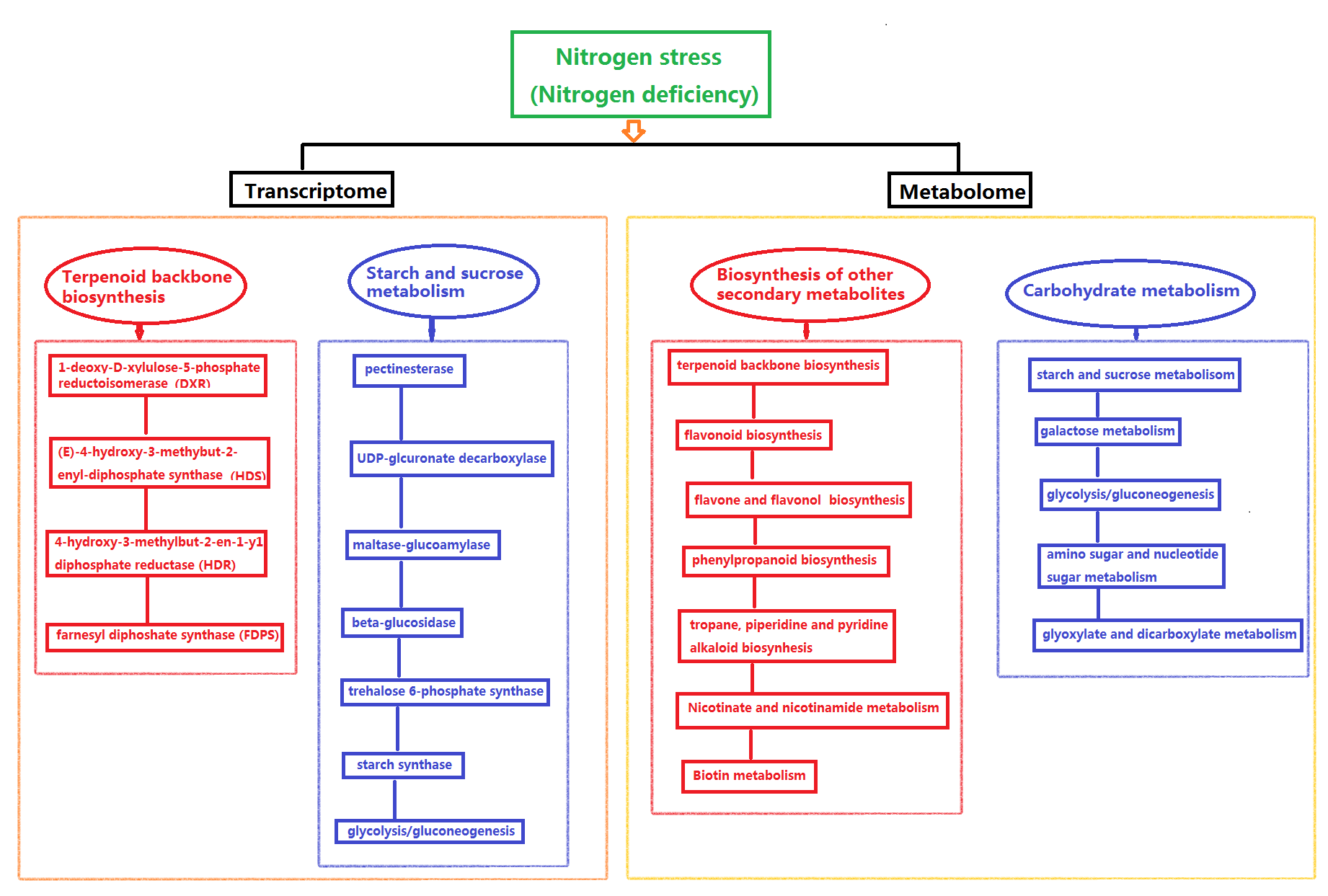

Supplement: S10 Fig — Red represents up-regulation and blue represents down-regulation. (TIF) [file pone.0273495.s010.tif]

Length Distribution of All-Unigene

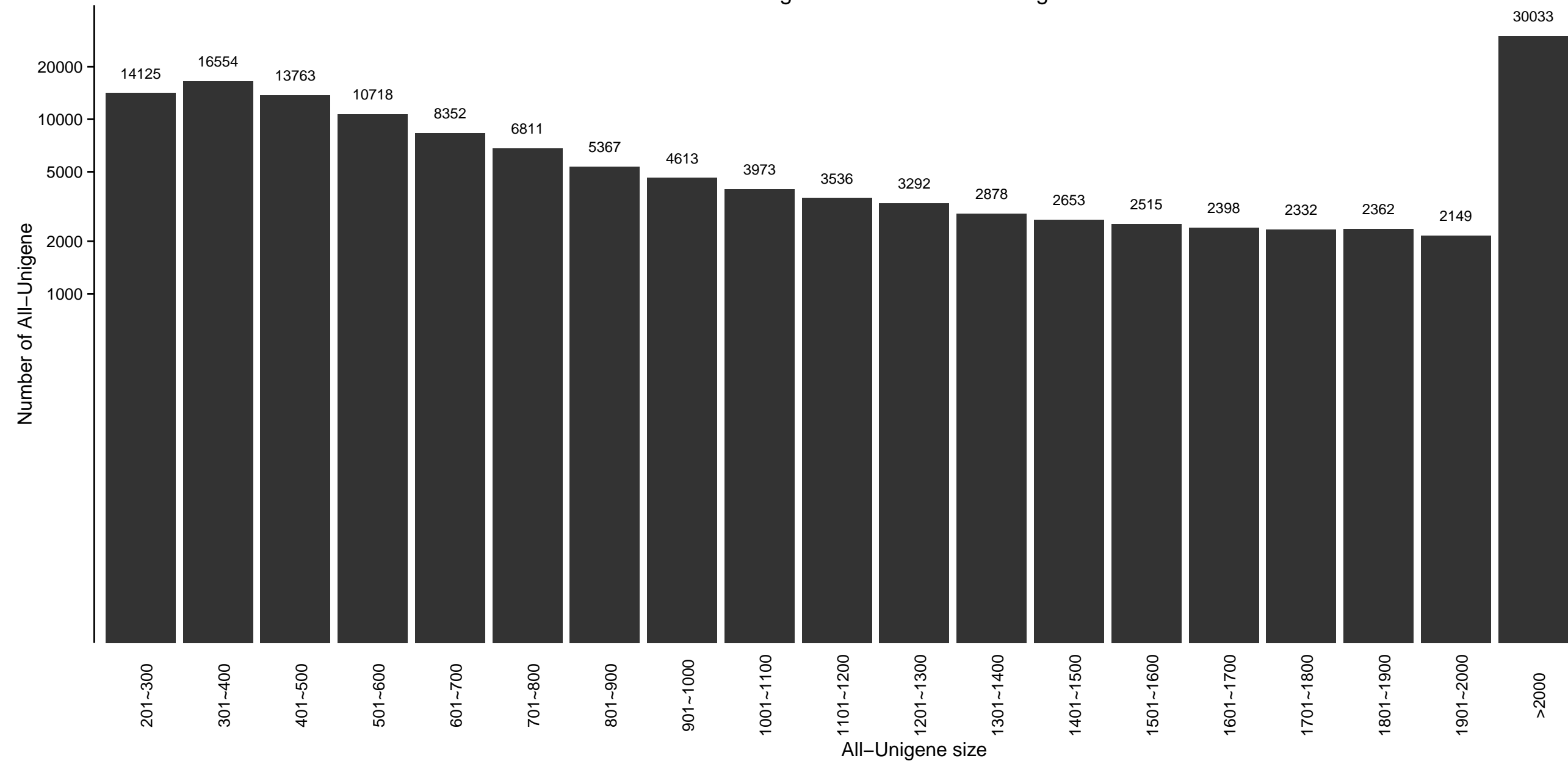

Supplement: S1 File — (PDF) [file pone.0273495.s023.pdf]

Unigene numbers

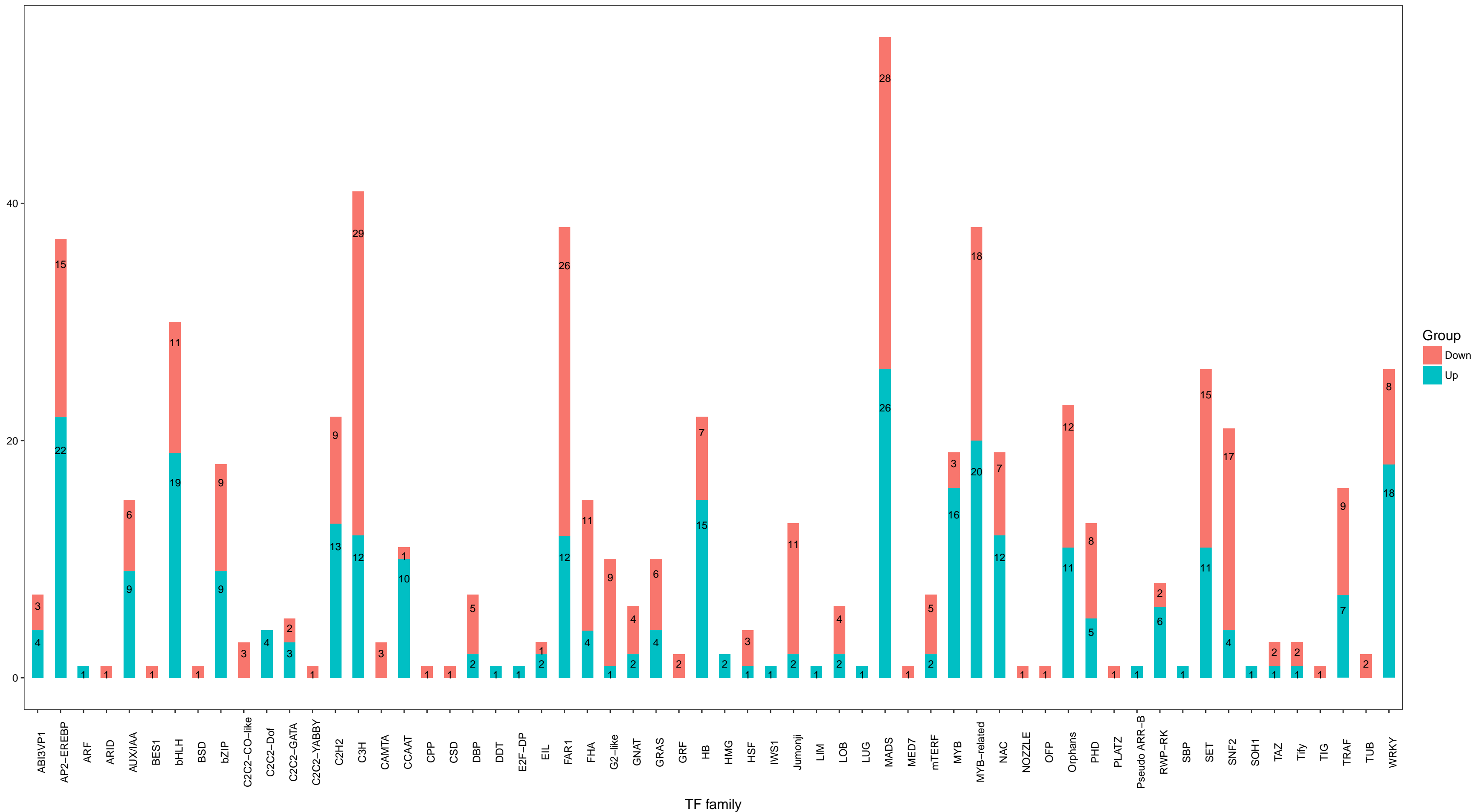

Supplement: S2 File — Transcription factor expression of the root of S. miltiorrhiza in N0 vs. Nf at 45 (a), 60(b) and 75 (c) days after transplanting (DAT). (d) TF family of the root of S. miltiorrhiza in the RNA-seq data. (ZIP) [file pone.0273495.s024.zip › S 8cú ̄ File.pdf]

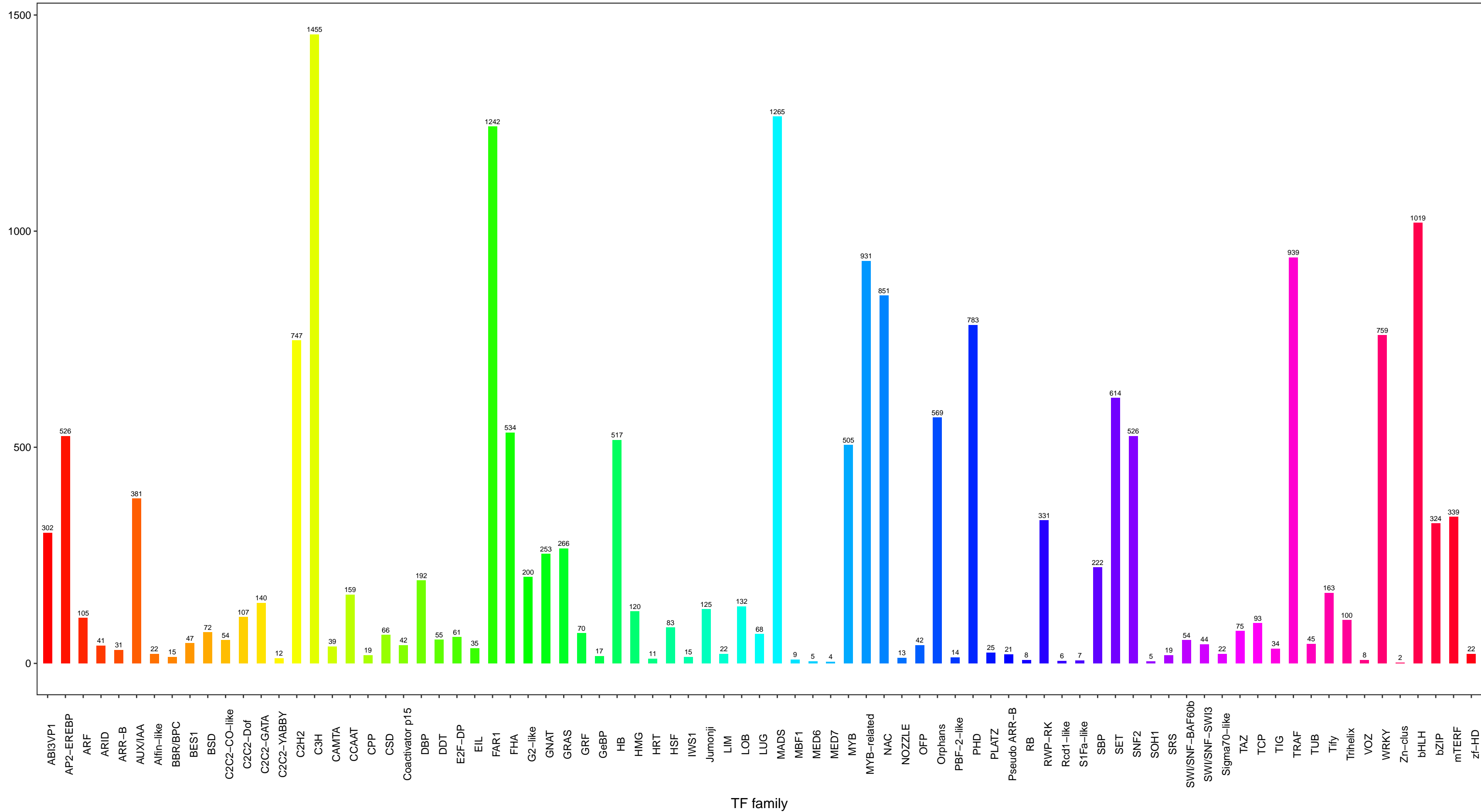

Supplement: S2 File — Transcription factor expression of the root of S. miltiorrhiza in N0 vs. Nf at 45 (a), 60(b) and 75 (c) days after transplanting (DAT). (d) TF family of the root of S. miltiorrhiza in the RNA-seq data. (ZIP) [file pone.0273495.s024.zip › S 8dú ̄File.pdf]

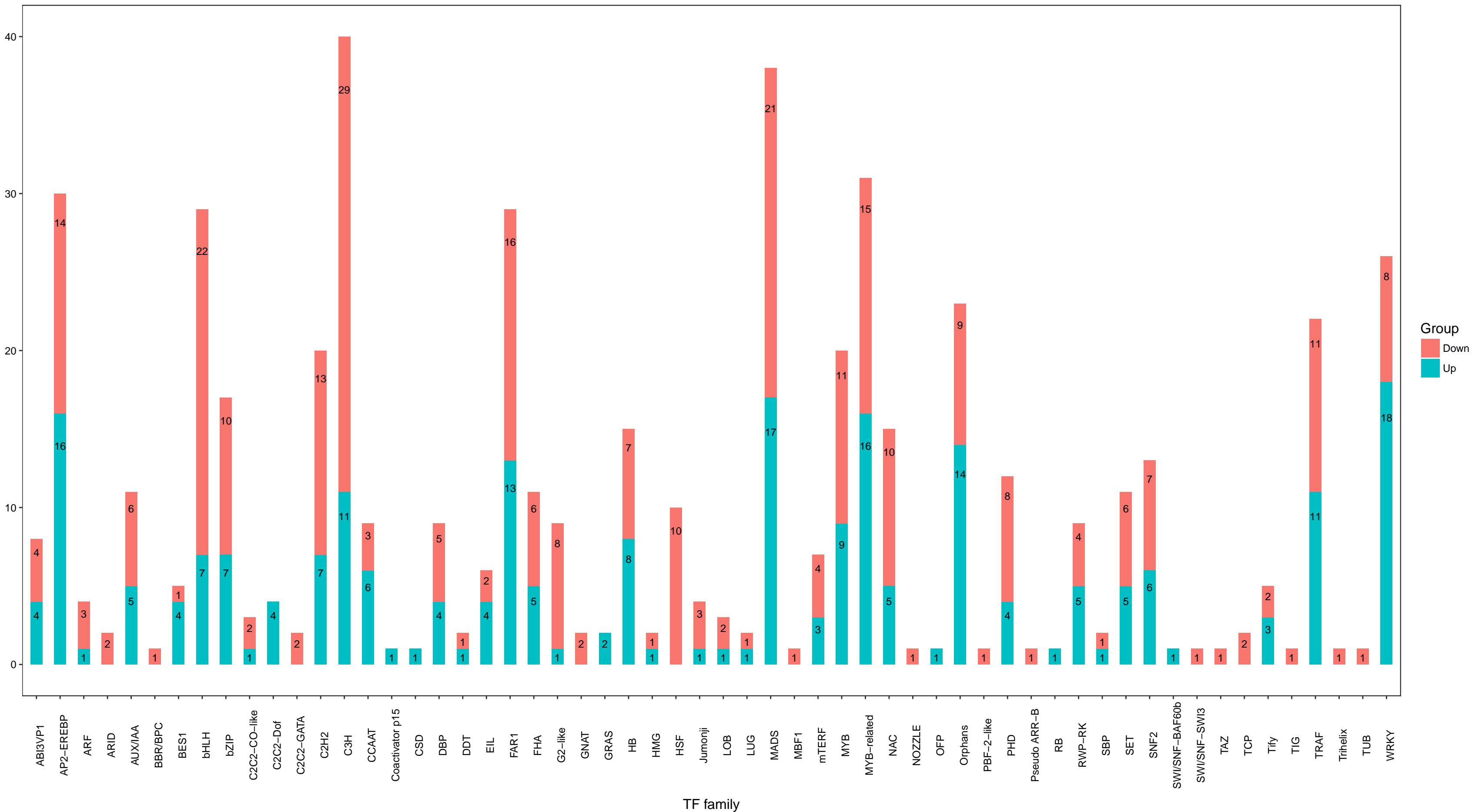

Supplement: S2 File — Transcription factor expression of the root of S. miltiorrhiza in N0 vs. Nf at 45 (a), 60(b) and 75 (c) days after transplanting (DAT). (d) TF family of the root of S. miltiorrhiza in the RNA-seq data. (ZIP) [file pone.0273495.s024.zip › S 8a ú ̄File.pdf]

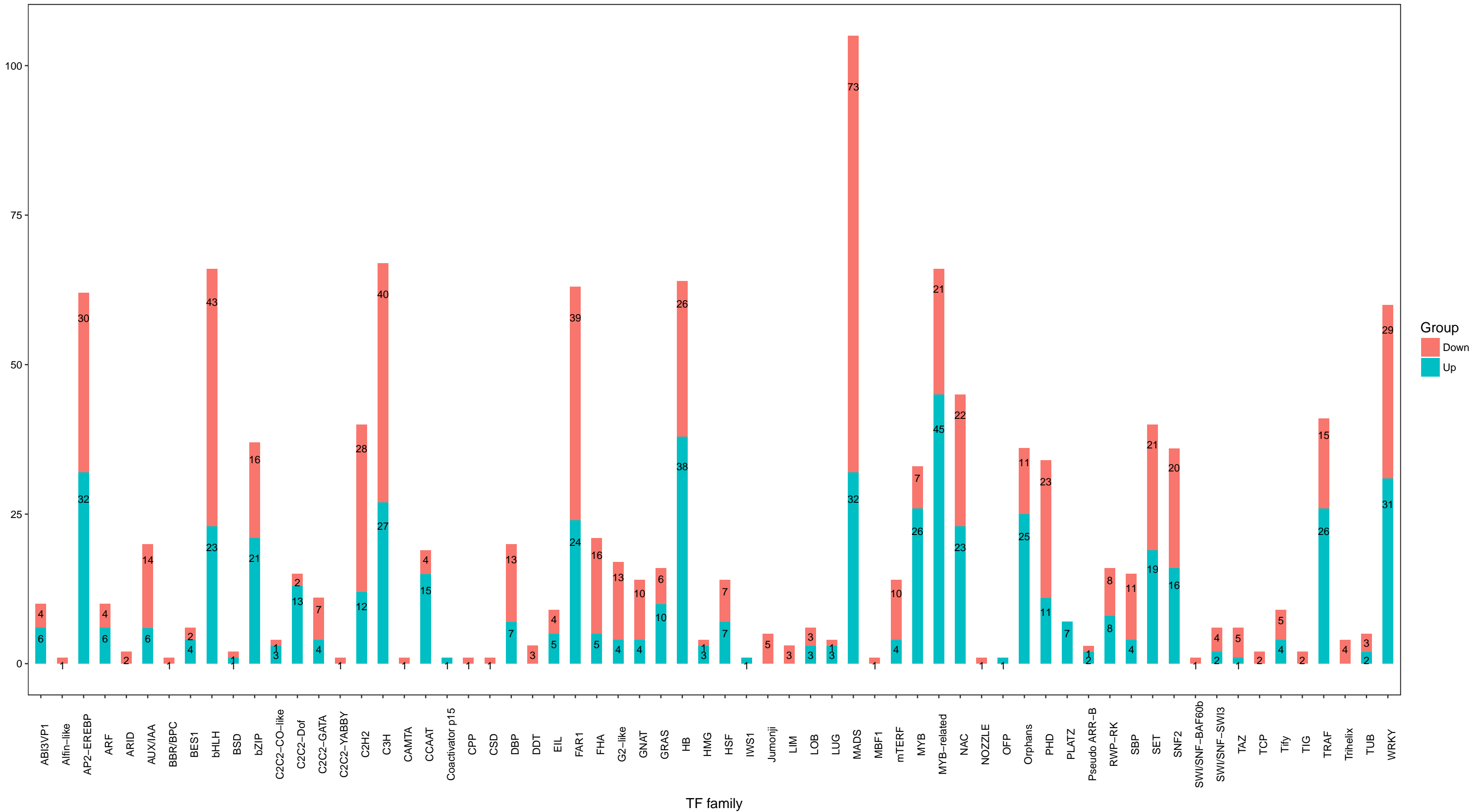

Supplement: S2 File — Transcription factor expression of the root of S. miltiorrhiza in N0 vs. Nf at 45 (a), 60(b) and 75 (c) days after transplanting (DAT). (d) TF family of the root of S. miltiorrhiza in the RNA-seq data. (ZIP) [file pone.0273495.s024.zip › S 8bú ̄File.pdf]

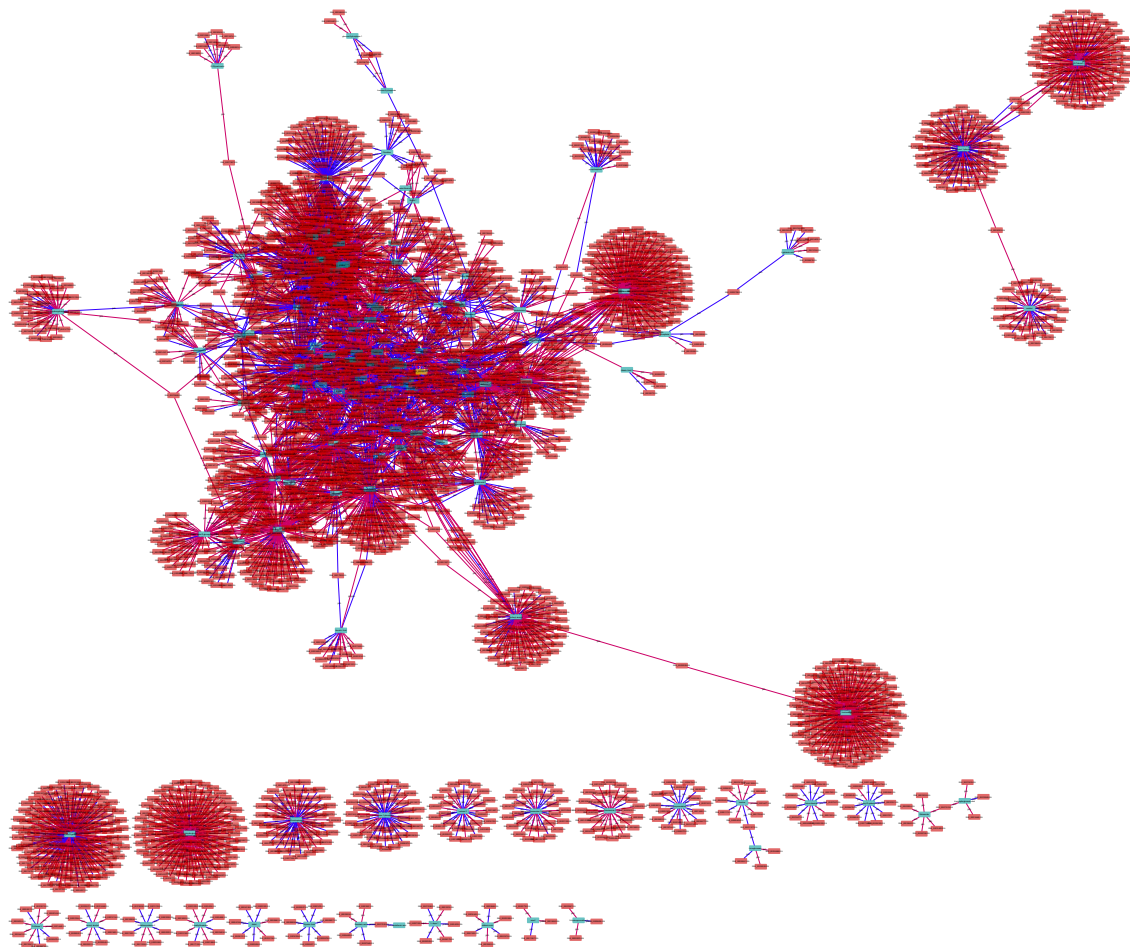

Supplement: S3 File — (PDF) [file pone.0273495.s025.pdf]
