# Supplementary material for: Integrative transcriptomic and metabolomic analyses unveil tanshinone biosynthesis in Salvia miltiorrhiza root under N starvation stress
Source: PLoS One. 2022 Aug 25;17(8):e0273495. doi: 10.1371/journal.pone.0273495 (PMC9409544; doi:10.1371/journal.pone.0273495)
Supplement: S2 Table — (DOCX) [file pone.0273495.s012.docx]

**S2 Table** Summary of the RNA-seq data collected from N0, Nl, and Nf at each of the three selected *S. miltiorrhiza* developmental stages. N0: no nitrogen; Nl: low nitrogen level; Nf: normal nitrogen level; DAT: days after transplanting

| **Sample name** |  | **N0** | **Nl** | **Nf** |
| --- | --- | --- | --- | --- |
| Raw reads number | 45 DAT | 70278140 | 70422780 | 70352670 |
|  | 60 DAT | 70293090 | 70465550 | 70279570 |
|  | 75 DAT | 70379020 | 70381390 | 70453830 |
|  | mean | 70316750 | 70423240 | 70362023 |
| Clean reads number | 45 DAT | 70278140 | 70422780 | 70352670 |
|  | 60 DAT | 70293090 | 70465550 | 70279570 |
|  | 75 DAT | 70379020 | 70381390 | 70453830 |
|  | mean | 70316750 | 70423240 | 70362023 |
| Total mapped number | 45 DAT | 67044795 (95.40%) | 67132181(95.32%) | 66986884(95.21%) |
|  | 60 DAT | 67167251 (95.55%) | 66606906(94.52%) | 67091585(95.46%) |
|  | 75 DAT | 67368603(95.72%) | 67058306 (95.27%) | 67115370(95.26%) |
|  | mean | 67193550 (95.56%) | 66932464(95.04%) | 67064613(95.31%) |
| Uniquely mapped reads | 45 DAT | 24298263(34.57%) | 24338172(34.56%) | 24293890(34.53%) |
|  | 60 DAT | 23324601(33.18%) | 23120446(32.81%) | 23371967(33.26%) |
|  | 75 DAT | 22902321(32.54%) | 22388454(31.81%) | 23889943(33.91%) |
|  | mean | 23508395(33.43%) | 23282357(33.06%) | 23851933(33.90%) |
| Multiple mapped reads | 45 DAT | 42746532(60.82%) | 42794009(60.77%) | 42692994(60.68%) |
|  | 60 DAT | 43842650 (62.37%) | 43486460(61.71%) | 43719618(62.21%) |
|  | 75 DAT | 44466283(63.18%) | 44669852(63.46%) | 43225427(61.35%) |
|  | mean | 43685155(62.12%) | 43650107(61.98%) | 43212680(61.41%) |
| Base number | 45 DAT | 8.78E+09 | 8.8E+09 | 8.79E+09 |
|  | 60 DAT | 8.79E+09 | 8.81E+09 | 8.78E+09 |
|  | 75 DAT | 8.8E+09 | 8.8E+09 | 8.81E+09 |
|  | mean | 8.79E+09 | 8.80E+09 | 8.79E+09 |
| GC content | 45 DAT | 48.00% | 47.50% | 49.00% |
|  | 60 DAT | 48.00% | 48.50% | 48.50% |
|  | 75 DAT | 48.00% | 48.00% | 48.50% |
|  | mean | 48.00% | 48.00% | 48.67% |
| Q30 | 45 DAT | 94.02% | 93.95% | 93.10% |
|  | 60 DAT | 94.32% | 93.43% | 93.67% |
|  | 75 DAT | 94.50% | 93.36% | 93.55% |
|  | mean | 94.28% | 93.58% | 93.44% |
