# Supplementary material for: Integrative transcriptomic and metabolomic analyses unveil tanshinone biosynthesis in Salvia miltiorrhiza root under N starvation stress
Source: PLoS One. 2022 Aug 25;17(8):e0273495. doi: 10.1371/journal.pone.0273495 (PMC9409544; doi:10.1371/journal.pone.0273495)
Supplement: S3 Table — (DOCX) [file pone.0273495.s013.docx]

**S3 Table**  Data of unigene length from *S. miltiorrhiza* mapped to the reference transcriptome.

|  | N50 | Max Length | Min Length | Average Length |
| --- | --- | --- | --- | --- |
| Unigene | 2197 | 28091 | 201 | 1296.57 |
