# Supplementary material for: Integrative transcriptomic and metabolomic analyses unveil tanshinone biosynthesis in Salvia miltiorrhiza root under N starvation stress
Source: PLoS One. 2022 Aug 25;17(8):e0273495. doi: 10.1371/journal.pone.0273495 (PMC9409544; doi:10.1371/journal.pone.0273495)
Supplement: S6 Table — (DOCX) [file pone.0273495.s016.docx]

**S6 Table** Data of GO enrichment DEGs statistics from *S. miltiorrhiza* mapped to the reference transcriptome in N0 vs. Nf, Nl vs. Nf, and N0 vs. Nf at 45, 60, and 75 days after transplanting (DAT)

| **DAT** | **Groups** | **Up/Down** | **Tested term** | **P value <0.05** | **P value <0.01** |
| --- | --- | --- | --- | --- | --- |
| 75 | Nf vs.N0 | up | 1422 | 764 | 585 |
|  | Nf vs.N0 | total | 2657 | 1045 | 597 |
|  | Nf vs.N0 | down | 1984 | 957 | 565 |
|  | Nf vs.Nl | up | 1256 | 678 | 548 |
|  | Nf vs.Nl | total | 1936 | 884 | 591 |
|  | Nf vs.Nl | down | 1237 | 685 | 537 |
|  | Nl vs.N0 | up | 727 | 472 | 405 |
|  | Nl vs.N0 | total | 2552 | 1289 | 915 |
|  | Nl vs.N0 | down | 2254 | 1255 | 998 |
| 60 | Nf vs.N0 | up | 1783 | 828 | 615 |
|  | Nf vs.N0 | total | 3358 | 1146 | 440 |
|  | Nf vs.N0 | down | 2620 | 1045 | 580 |
|  | Nf vs.Nl | up | 918 | 613 | 533 |
|  | Nf vs.Nl | total | 2159 | 972 | 668 |
|  | Nf vs.Nl | down | 1721 | 831 | 568 |
|  | Nl vs.N0 | up | 1078 | 616 | 524 |
|  | Nl vs.N0 | total | 2943 | 1373 | 867 |
|  | Nl vs.N0 | down | 2488 | 1343 | 964 |
| 45 | Nf vs.N0 | up | 1394 | 769 | 598 |
|  | Nf vs.N0 | total | 2200 | 957 | 606 |
|  | Nf vs.N0 | down | 1471 | 718 | 496 |
|  | Nf vs.Nl | up | 941 | 534 | 434 |
|  | Nf vs.Nl | total | 1516 | 749 | 492 |
|  | Nf vs.Nl | down | 982 | 572 | 459 |
|  | Nl vs.N0 | up | 317 | 217 | 201 |
|  | Nl vs.N0 | total | 647 | 394 | 343 |
|  | Nl vs.N0 | down | 443 | 306 | 288 |
