# Supplementary material for: Integrative transcriptomic and metabolomic analyses unveil tanshinone biosynthesis in Salvia miltiorrhiza root under N starvation stress
Source: PLoS One. 2022 Aug 25;17(8):e0273495. doi: 10.1371/journal.pone.0273495 (PMC9409544; doi:10.1371/journal.pone.0273495)
Supplement: S8 Table — (DOCX) [file pone.0273495.s018.docx]

**S8 Table** Data of KEGG enrichment pathways statistics from *S. miltiorrhiza* mapped to the reference transcriptome in N0 vs. Nf, Nl vs. Nf, and N0 vs. Nl at 45, 60, and 75 days after transplanting (DAT)

| **DAT** | **Groups** | **Up/Down** | **Tested term** | **P Value <0.05** | **P Value <0.01** |
| --- | --- | --- | --- | --- | --- |
| 75 | Nf vs.N0 | up | 138 | 23 | 15 |
|  | Nf vs.N0 | total | 174 | 28 | 14 |
|  | Nf vs.N0 | down | 156 | 41 | 17 |
|  | Nf vs.Nl | up | 131 | 29 | 19 |
|  | Nf vs.Nl | total | 156 | 33 | 19 |
|  | Nf vs.Nl | down | 131 | 28 | 13 |
|  | Nl vs.N0 | up | 105 | 35 | 14 |
|  | Nl vs.N0 | total | 168 | 39 | 19 |
|  | Nl vs.N0 | down | 156 | 42 | 20 |
| 60 | Nf vs.N0 | up | 140 | 29 | 22 |
|  | Nf vs.N0 | total | 186 | 32 | 21 |
|  | Nf vs.N0 | down | 172 | 35 | 15 |
|  | Nf vs.Nl | up | 80 | 31 | 15 |
|  | Nf vs.Nl | total | 163 | 34 | 21 |
|  | Nf vs.Nl | down | 157 | 44 | 20 |
|  | Nl vs.N0 | up | 115 | 35 | 25 |
|  | Nl vs.N0 | total | 176 | 43 | 28 |
|  | Nl vs.N0 | down | 168 | 42 | 22 |
| 45 | Nf vs.N0 | up | 146 | 44 | 25 |
|  | Nf vs.N0 | total | 175 | 46 | 33 |
|  | Nf vs.N0 | down | 152 | 31 | 16 |
|  | Nf vs.Nl | up | 114 | 36 | 14 |
|  | Nf vs.Nl | total | 152 | 36 | 18 |
|  | Nf vs.Nl | down | 123 | 33 | 14 |
|  | Nl vs.N0 | up | 46 | 25 | 6 |
|  | Nl vs.N0 | total | 78 | 27 | 9 |
|  | Nl vs.N0 | down | 57 | 28 | 10 |
