# Supplementary material for: Integrative transcriptomic and metabolomic analyses unveil tanshinone biosynthesis in Salvia miltiorrhiza root under N starvation stress
Source: PLoS One. 2022 Aug 25;17(8):e0273495. doi: 10.1371/journal.pone.0273495 (PMC9409544; doi:10.1371/journal.pone.0273495)
Supplement: S10 Table — (DOCX) [file pone.0273495.s020.docx]

**S10 Table** Tanshinone Synthetic(Terpenoid Backbone Biosynthesis) (KEGG) Pathway Gene Expression in N0 vs. Nf and Nl vs. Nf at 75 days after transplanting (DAT).

**(a) in N0 vs. Nf at 75DAT**

| **Enzyme** | **Gene name** | **KO；GI** | **FC(N0/Nf)** |
| --- | --- | --- | --- |
| [EC: 2.2.1.7] | 1-deoxy-D-xylulose-5-phosphate synthase (DXS) | CL1282Contig1  CL1282Contig2  Comp63666-c1-seq3-9 | 0.3585  0.1733  10.0368 |
| [EC: 1.1.1.267] | 1-deoxy-D-xylulose-5-phosphate reductoisomerase (DXR) | CL589Contig4  Comp54258-c0-seq3-4 | 1.6412  1.7476 |
| [EC: 1.17.7.1] | (E)-4-hydroxy-3-methylbut-2-enyl-diphosphate synthase(HDS) | Comp59962-c0-seq1-4 | 1.5322 |
| [EC: 1.17.7.2] | 4-hydroxy-3-methylbut-2-en-1-yl diphosphate reductase(HDR/IDS) | CL540Contig1  Comp47624-c0-seq1-4 | 1.6465  1.3614 |
| [EC: 2.3.1.9] | acetyl-CoA C-acetyltransferase(AACT) | CL10790Contig1 | 1.4877 |
| [EC: 2.3.3.10] | hydroxymethylglutaryl-CoA synthase (HMGS) | Comp60047-c1-seq14-6 | 1.5209 |
| [EC: 1.1.1.34] | hydroxymethylglutaryl-CoA reductase (HMGR) | CL28921Contig1  CL35976Contig1 | 2.5930  2.5900 |
| [EC: 2.7.1.36] | mevalonate kinase（MK） | CL16608Contig1 | 1.4495 |
| [EC: 2.5.1.1] | geranyl diphosphate synthase(GPS) | CL4842Contig1  CL8090Contig1 | 1.4143  1.7523 |
| [EC: 2.5.1.10] | farnesyl diphosphate synthase (FPS) | CL4842Contig1  CL8090Contig1 | 1.4143  1.7523 |
| [EC: 2.5.1.29] | geranylgeranyl diphosphate synthase, type II (GGPS) | CL8090Contig1 | 1.7523 |
| [EC: 2.1.1.100] | protein-S-isoprenylcysteine O-methyltransferase (ICMT) | Comp45317-c0-seq4-1 | 4.1729 |

**(b) in Nl vs. Nf at 75DAT**

| Enzyme | Gene name | GI | FC(Nl/Nf) |
| --- | --- | --- | --- |
| [EC: 2.2.1.7] | 1-deoxy-D-xylulose-5-phosphate synthase (DXS) | - | - |
| [EC: 1.1.1.267] | 1-deoxy-D-xylulose-5-phosphate reductoisomerase (DXR) | CL589Contig4  Comp54258-c0-seq3-4 | 1.9083  2.5386 |
| [EC: 1.17.7.1] | (E)-4-hydroxy-3-methylbut-2-enyl-diphosphate synthase(HDS) | CL870Contig2 | 1.4646 |
| [EC: 1.17.7.2] | 4-hydroxy-3-methylbut-2-en-1-yl diphosphate reductase(HDR/IDS) | CL540Contig1  Comp47624-c0-seq1-4 | 1.8624  1.4642 |
| [EC: 2.3.1.9] | acetyl-CoA C-acetyltransferase(AACT) | - | - |
| [EC: 2.3.3.10] | hydroxymethylglutaryl-CoA synthase (HMGS) | - | - |
| [EC: 1.1.1.34] | hydroxymethylglutaryl-CoA reductase (HMGR) | - | - |
| [EC: 2.7.1.36] | mevalonate kinase（MK） | - | - |
| [EC: 2.5.1.1] | geranyl diphosphate synthase(GPS) | CL4842Contig1  Comp57112-c1-seq1-6  CL116Contig1  CL8090Contig1  Comp64888-c0-seq8-5 | 1.3532  2.6941  0.5613  2.3460  4.6125 |
| [EC: 2.5.1.10] | farnesyl diphosphate synthase (FPS) | CL4842Contig1  Comp57112-c1-seq1-6  CL116Contig1  CL8090Contig1  Comp64888-c0-seq8-5 | 1.3532  2.6941  0.5613  2.3460  4.6125 |
| [EC: 2.5.1.29] | geranylgeranyl diphosphate synthase, type II (GGPS) | CL116Contig1  CL8090Contig1  Comp64888-c0-seq8-5 | 0.5613  2.3460  4.6125 |
| [EC: 2.1.1.100] | protein-S-isoprenylcysteine O-methyltransferase (ICMT) | - | - |
