# Supplementary material for: Integrative transcriptomic and metabolomic analyses unveil tanshinone biosynthesis in Salvia miltiorrhiza root under N starvation stress
Source: PLoS One. 2022 Aug 25;17(8):e0273495. doi: 10.1371/journal.pone.0273495 (PMC9409544; doi:10.1371/journal.pone.0273495)
Supplement: S11 Table — (DOCX) [file pone.0273495.s021.docx]

**S11 Table  OPLS-DA model cumulative interpretation rate**

| **Model** | **Type** | **A** | **N** | **R2X(cum)** | **R2Y(cum)** | **Q2 (cum)** | **Title** |
| --- | --- | --- | --- | --- | --- | --- | --- |
| M1 | OPLS-DA | 1+1+0 | 10 | 0.313 | 0.972 | 0.452 | N1(45d) VS N1(60d) |
| M2 | OPLS-DA | 1+1+0 | 10 | 0.361 | 0.964 | 0.399 | N1(60d) VS N1(75d) |
| M3 | OPLS-DA | 1+1+0 | 10 | 0.346 | 0.987 | 0.625 | N1(45d) VS N1(75d) |
| M4 | OPLS-DA | 1+1+0 | 10 | 0.328 | 0.963 | 0.194 | N1(45d) VS N0(45d) |
| M5 | OPLS-DA | 1+1+0 | 10 | 0.402 | 0.957 | 0.207 | N1(60d) VS N0(60d) |
| M6 | OPLS-DA | 1+1+0 | 10 | 0.295 | 0.977 | 0.177 | N1(75d) VS N0(75d) |
| M7 | OPLS-DA | 1+1+0 | 10 | 0.342 | 0.995 | 0.772 | N1(45d) VS Nf(45d) |
| M8 | OPLS-DA | 1+1+0 | 10 | 0.369 | 0.993 | 0.853 | N1(60d) VS Nf(60d) |
| M9 | OPLS-DA | 1+1+0 | 10 | 0.402 | 0.994 | 0.765 | N1(75d) VS Nf(75d) |

Note: R^2^Y (representing the interpretability of the Y variable), Q2 (predictability of the model), which were closer to 1 indicates that the OPLS-DA model better explains the difference between the two groups of samples.
